# Meta-Analysis: Shouldn’t Prophylactic Corticosteroids be Administered During Cardiac Surgery with Cardiopulmonary Bypass?

**DOI:** 10.3389/fsurg.2022.832205

**Published:** 2022-06-01

**Authors:** Tianci Chai, Xinghui Zhuang, Mengyue Tian, Xiaojie Yang, Zhihuang Qiu, Shurong Xu, Meiling Cai, Yanjuan Lin, Liangwan Chen

**Affiliations:** ^1^ Department of Cardiovascular Surgery, Fujian Medical University Union Hospital, Fuzhou, China; ^2^ Key Laboratory of Ministry of Education for Gastrointestinal Cancer, The School of Basic Medical Sciences, Fujian Medical University, Fuzhou, China; ^3^ Key Laboratory of Cardio-Thoracic Surgery (Fujian Medical University), Fujian Province University, Fuzhou, China; ^4^ Department of anesthesiology, Xinyi People’s Hospital, Xuzhou, China; ^5^ Department of Thoracic Surgery, Fujian Medical University Union Hospital, Fuzhou, China; ^6^ Nursing Department, Fujian Medical University Union Hospital, Fuzhou, China

**Keywords:** corticosteroids prophylaxis, cardiac surgery, cardiopulmonary bypass, myocardial infarction, randomized controlled trial

## Abstract

**Background:**

Corticosteroids can effectively inhibit systemic inflammation induced by cardiopulmonary bypass. Recently clinical trials and meta-analyses and current guidelines for cardiac surgery do not support corticosteroids prophylaxis during cardiac surgery because of an increase in myocardial infarction and no benefit for patients. The aim of this study is to determine whether specific corticosteroids dose ranges might provide clinical benefits without increasing myocardial infarction.

**Methods:**

The PubMed, Web of Science, Embase, Clinical Trials, and Cochrane databases were searched for randomized controlled trials (RCTs) published before August 1, 2021.

**Results:**

88 RCTs with 18,416 patients (17,067 adults and 1,349 children) were identified. Relative to placebo and high-dose corticosteroids, low-dose corticosteroids (≤20 mg/kg hydrocortisone) during adult cardiac surgery did not increase the risks of myocardial infarction (odds ratio [OR]: 0.96, 95% confidence interval [CI]: 0.43–2.17; *p* = 0.93). However, low-dose corticosteroids were associated with lower risks of atrial fibrillation (OR: 0.58, 95% CI: 0.44–0.76; *p* < 0.0001) and kidney injury (OR: 0.29, 95% CI: 0.09–0.96; *p* = 0.04). Furthermore, low-dose corticosteroids significantly shortened the mechanical ventilation times (mean difference [MD]: −2.74 h, 95% CI: −4.14, −1.33; *p* = 0.0001), intensive care unit (ICU) stay (MD: −1.48 days, 95% CI: −2.73, −0.22; *p* = 0.02), and hospital stay (MD: −2.29 days, 95% CI: −4.51, −0.07; *p* = 0.04).

**Conclusion:**

Low-dose corticosteroids prophylaxis during cardiac surgery provided significant benefits for adult patients, without increasing the risks of myocardial infarction and other complications.

## Introduction

Cardiopulmonary bypass (CPB) is used during most cardiac surgeries, although CPB often induces systemic inflammatory response syndrome (SIRS) (1). The development of SIRS involves activation of complement, platelets, neutrophils, monocytes, macrophages, and cascade reactions, which leads to increased endothelial permeability, blood vessel damage, and parenchymal cell damage (2–4). These events are associated with single and multiple organ dysfunction, myocardial injury and infarction, respiratory failure, and ultimately death (5–7).

Corticosteroids are inexpensive drugs that can effectively inhibit inflammation, limit systemic capillary leak syndrome, and reduce organ damage, which provides a theoretical basis for their use during CPB (8–10). However, corticosteroids can cause side effects, including hyperglycemia, which is associated with immunosuppression and poor wound healing (11, 12). In addition, high-dose corticosteroids are associated with an increased risk of gastrointestinal bleeding and myocardial infarction (11, 12). Thus, the benefits of corticosteroids treatment are controversial for patients undergoing cardiac surgery with CPB (13–15).

Three meta-analyses of small RCTs revealed that prophylactic corticosteroids could reduce the risk of atrial fibrillation after adult cardiac surgery, also caused some side effects (5–7). Two large multi-center RCTs subsequently revealed that corticosteroids therapy provided no benefits and increased the risk of myocardial infarction in adult patients (13, 14). Thus, the adult cardiac surgery guidelines do not recommend routine prophylactic use of corticosteroids during cardiac surgery (16), although there are no specific guidelines regarding corticosteroids use during pediatric cardiac surgery. We hypothesized that the specific corticosteroids dose range might influence the risks and benefits during cardiac surgery with CPB. Therefore, this systematic review and meta-analysis aimed to evaluate the dose-dependent benefits and risks of prophylactic corticosteroids for adults and children undergoing cardiac surgery with CPB.

## Methods

### Ethical Statement

This study was a meta-analysis of the results of published randomized controlled trials, and ethical approval and informed consent of patients were not required.

### Search Strategy and Selection Criteria

Two authors (XJY and MYT) searched the PubMed, Web of Science, Embase, ClinicalTrials, and Cochrane Central Register of Controlled Trials databases for relevant RCTs that were published in any language before August 1, 2021. The reference lists of relevant articles were also manually checked. The study protocol followed the PRISMA-P guidelines (https://www.crd.york.ac.uk/prospero/display_record.php?ID=CRD42020193658). The search terms were: (“corticosteroids” OR “dexamethasone” OR “prednisolone” OR “prednisone” OR “methylprednisolone” OR “hydrocortisone”) AND (“cardiopulmonary bypass” OR “cardiac surgery”) AND (“randomized controlled trials”) (Supplementary Table S1).

The meta-analysis only included RCTs that compared corticosteroids with a placebo used before or at the beginning of CPB. Patients undergoing surgery with CPB for heart and/or valvular diseases were included. And the studies were excluded if they used different concomitant medications or evaluated corticosteroids during off-pump heart surgery.

Two authors (TCC and XHZ) independently determined whether the identified articles fulfilled the inclusion criteria. The two authors also independently used pre-designed data extraction forms to record information regarding trial characteristics, clinical outcomes, randomization methods, application of blinding, allocation concealment, inclusion criteria, and exclusion criteria. There were no instances of disagreement regarding the extracted data.

### Data Analysis

Study characteristics included first author, publication date, country, study size, study design, randomization, blinding, follow-up duration, patient withdrawals, and study duration. Patient characteristics included age, sex, surgery type, blood pressure, history of diabetes, history of smoking, renal status, and fulfillment of the inclusion criteria. The interventions included the corticosteroids type, dose, timing, and route of administration during CPB.

The primary outcomes included myocardial infarction, insulin use, mortality, new atrial fibrillation, lengths of ICU and hospital stays, acute kidney injury, mechanical ventilation time. The secondary outcomes included postoperative bleeding, re-intubation, duration of CPB and procedure, pulmonary complications (pulmonary edema), postoperative infection, neurological complications (stroke), delirium, gastrointestinal bleeding, extracorporeal membrane oxygenation (ECMO) use, vasoactive medication use, re-thoracotomy, inotropic score, blood transfusion, and and blood concentrations of glucose, lactate, C-reactive protein (CRP), tumor necrosis factor-α(TNF-α), interleukin (IL)-6, IL-8, and IL-10 at 24 h after CPB.

The Cochrane Handbook for Systematic Reviews of Interventions and Jadad score were used to assess the risk of bias for each trial (17, 18). The data were synthesized using Review Manager version 5.3 and Stata software version 16 (StataCorp, College Station, TX, USA). Inter-study heterogeneity was assessed using the chi-squared test and *I*^2^ statistic, with the random effects model used for data with high heterogeneity (*p *< 0.1 or *I*^2^ > 50%) and the fixed effects model used for data with less heterogeneity. The Mantel-Haenszel method was used to pool binary data and the results were reported as ORs with 95% CIs. An inverse variance analysis method was used to pool continuous data and the results were reported as MDs with 95% CIs. The GRADEpro GDT (https://www.gradepro.org/) was used to classify the certainty of evidence.

## Results

**Figure 1:**
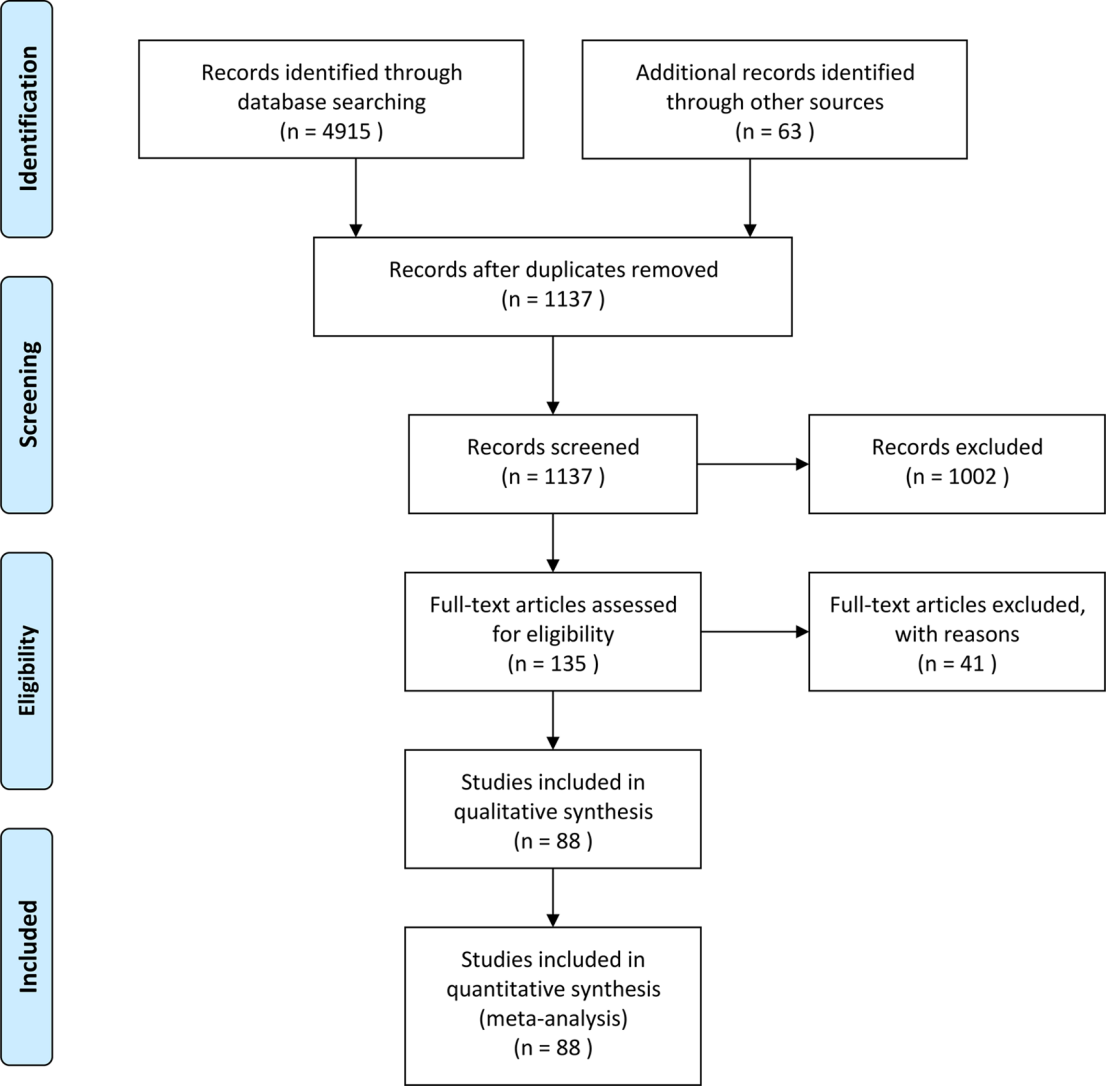
PRISMA flow diagram.

This study identified 88 RCTs from 27 countries that included 18,416 patients (Figure 1). The trials considered adult patients (73 trials and 17,067 patients) or pediatric patients (15 trials and 1,349 patients). The corticosteroids treatments included dexamethasone, betamethasone, methylprednisolone, hydrocortisone, and prednisolone, with a broad range of total doses (1–900 mg/kg hydrocortisone equivalent). Table 1 shows the characteristics of the included studies, Table 2 shows the GRADE summary of the findings and Table 3 summarizes the impact of corticosteroids on adults and pediatric.

**Table 1:** Characteristics of the included studies.

	Country	Population 0000 size (*n*)	Patient population	Age group	Study design	Blinding	Follow-up	Steroid	Hydrocortisone equivalent dose (mg/kg)	Time of administration	Quality^a^
Abbaszadeh et al. (2012) (24)	Iran	185	CABG	Adults	RCT	Y	3 days	Dexamethasone	4.6	Post induction and surgery	High (7)
Abd El-Hakeem et al. (2003a) (25)	Egypt	46	Valve	Adults	RCT	Y	ICU stay	Dexamethasone	38	Pre CPB	High (7)
Abd El-Hakeem et al. (2003b) (25)	Egypt	20	Valve	Adults	RCT	Y	ICU stay	Dexamethasone	38	Pre CPB	High (7)
Al-Shawabkeh et al. (2016) (26)	Jordan	340	CABG or valve	Adults	RCT	Y	96 h	Methylprednisolone and hydrocortisone	84	Pre CPB and last for 3 days	High (7)
Amanullah et al. (2016) (27)	Pakistan	129	Cardiac surgery	Children	RCT	Y	Hospital stay	Dexamethasone	80	Pre CPB and post surgery	High (7)
Andersen et al. (1989) (28)	Denmark	16	CABG	Adults	RCT	N	7 days	Methylprednisolone	150	Pre CPB	Low (1)
Ando et al. (2005) (29)	Japan	20	Cardiac surgery	Children	RCT	Y	Hospital stay	Hydrocortisone	17.71	Post CPB	High (6)
Bingol et al. (2005) (30)	Turkey	40	CABG	Adults	RCT	Y	3 months	Prednisolone	11	Pre induction and post surgery	High (6)
Boscoe et al. (1983) (31)	UK	34	CABG or valve or complex	Adults	RCT	Unclear	24 h	Methylprednisolone	300	Pre CPB	Low (3)
Bourbon et al. (2004) (32)	France	36	CABG	Adults	RCT	N	24 h	Methylprednisolone	25/50	Pre CPB	Low (2)
Brettner et al. (2019) (33)	Germany	30	Cardiac surgery	Adults	RCT	Y	28 days	Hydrocortisone	1	Pre surgery	High (4)
Bronicki et al. (2000) (34)	US	29	Cardiac surgery	Children	RCT	Y	Hospital stay	Dexamethasone	26.67	Pre CPB	High (6)
Butler et al. (1996) (35)	UK	18	Cardiac surgery	Children	RCT	Y	Hospital stay	Methylprednisolone	50	During initiation of CPB	High (6)
Cavarocchi et al. (1986) (36)	US	61	CABG or valve or complex	Adults	RCT	N	24 h	Methylprednisolone	150	Pre CPB	Low (2)
Celik et al. (2004) (37)	Turkey	60	CABG	Adults	RCT	Y	Hospital stay	Methylprednisolone	900	Pre surgery	High (4)
Chaney et al. (1998/1999) (38, 39)	US	60	CABG	Adults	RCT	Y	Hospital stay	Methylprednisolone	300	During sternotomy and pre CPB	High (4)
Chaney et al. (2001) (40)	US	90	CABG	Adults	RCT	Y	Hospital stay	Methylprednisolone	300/150	During sternotomy and initiation of CPB	High (4)
Checchia et al. (2003) (41)	US	28	Cardiac surgery	Children	RCT	Y	Hospital stay	Dexamethasone	26.67	Pre CPB	High (6)
Codd et al. (1977) (42)	US	150	CABG	Adults	RCT	Unclear	5 days	Methylprednisolone	143	Pre CPB	Low (3)
Coetzer et al. (1996) (43)	South Africa	295	Cardiac surgery	Adults	RCT	Unclear	30 days	Methylprednisolone	150	Pre CPB	High (5)
Danielson et al. (2018) (44)	Sweden	30	CABG or valve	Adults	RCT	Y	Hospital stay	Methylprednisolone	75	Post induction	High (5)
Demir et al. (2009) (45)	Turkey	30	CABG	Adults	RCT	Unclear	Hospital stay	Methylprednisolone	143	Pre CPB	Low (3)
Demir et al. (2015) (46)	Turkey	40	CABG	Adults	RCT	Y	Unclear	Methylprednisolone	71	Pre CPB	Low (3)
Dieleman et al. (2012) (13)	Netherlands	4494	CABG or valve or complex	Adults	RCT	Y	12 months	Dexamethasone	27	Pre CPB	High (7)
El Azab et al. (2002) (47)	Netherlands	18	CABG	Adults	RCT	Y	Hospital stay	Dexamethasone	38	Pre surgery	High (5)
Enc et al. (2006) (48)	Turkey	40	CABG	Adults	RCT	Y	Hospital stay	Methylprednisolone	125	Pre CPB	High (6)
Engelman et al. (1995) (49)	US	19	CABG	Adults	RCT	Y	Hospital stay	Methylprednisolone and dexamethasone	78	Pre CPB and post surgery	High (4)
Fecht et al. (1978) (50)	US	50	CABG	Adults	RCT	Y	Hospital stay	Methylprednisolone	286	Pre CPB	High (4)
Ferries et al. (1984/1987) (51)	US	80	CABG or valve or complex	Adults	RCT	Y	Hospital stay	Methylprednisolone	150	Pre CPB	High (5)
Fillinger et al. (2002) (52)	US	50	CABG	Adults	RCT	Y	Hospital stay	Methylprednisolone	81	Pre incision and post surgery	High (7)
Giomarelli et al. (2003) (53)	Italy	20	CABG	Adults	RCT	Y	Hospital stay	Methylprednisolon	116	Pre surgery and post CPB	High (7)
Gomez et al. (2018) (54)	Spain	104	CABG or valve	Adults	RCT	Y	Unclear	Methylprednisolone and dexamethasone	40	Post induction and surgery	High (4)
Graham et al. (2019) (55)	US	176	Cardiac surgery	Children	RCT	Y	90 days	Methylprednisolone	150	Post induction	High (7)
Halonen et al. (2007) (56)	Finland	241	CABG or valve	Adults	RCT	Y	ICU stay	Hydrocortisone	14	Pre surgery	High (7)
Halvorsen et al. (2003) (57)	US	300	CABG	Adults	RCT	Y	ICU stay	Dexamethasone	3	Post induction	High (6)
Hao et al. (2019) (58)	China	36	Valve	Adults	RCT	Y	Hospital stay	Methylprednisolone	36	During initiation of CPB	Low (3)
Harig et al. (1999/2001) (59, 60)	Germany	40	CABG	Adults	RCT	N	30 days	Prednisolone	29	Pre induction and post surgery	Low (2)
Heying et al. (2012) (61)	Germany	20	Cardiac surgery	Children	RCT	Y	Hospital stay	Dexamethasone	26.67	Pre CPB	High (6)
Jansen et al. (1991) (62)	Netherlands	25	CABG	Adults	RCT	Y	Hospital stay	Dexamethasone	27	Pre CPB	High (4)
Keski-Nisula et al. (2013) (15)	Finland	40	Cardiac surgery	Children	RCT	Y	Hospital stay	Methylprednisolone	150	Post induction	High (6)
Keski-Nisula et al. (2015) (63)	Finland	45	Cardiac surgery	Children	RCT	Y	Hospital stay	Methylprednisolone	150	During induction and initiation of CPB	High (6)
Keski-Nisula et al. (2020) (64)	Finland	29	Cardiac surgery	Children	RCT	Y	Hospital stay	Methylprednisolone	150	During induction	High (6)
Kilger Schelling et al. (2003/2004) (65, 66)	Germany	91	CABG or valve	Adults	RCT	N	6 months	Hydrocortisone	8	Pre induction and last for 3 days	Low (3)
Kilickan et al. (2008) (67)	Turkey	60	CABG	Adults	RCT	N	Hospital stay	Methylprednisolone	75	Pre induction	Low (3)
Liakopoulos et al. (2007) (68)	Germany	78	CABG	Adults	RCT	Y	Hospital stay	Methylprednisolone	75	Pre CPB	High (4)
Lindberg et al. (2003) (69)	Sweden	40	Cardiac surgery	Children	RCT	Y	Hospital stay	Dexamethasone	26.67	During surgery	High (6)
Loef et al. (2004) (4)	Netherlands	20	CABG	Adults	RCT	Y	Hospital stay	Dexamethasone	40	Pre induction and post surgery	High (4)
Lomivorotov et al. (2013) (70)	Russia	50	CABG	Adults	RCT	Y	24 h	Methylprednisolone	100	Post induction	High (4)
Lomivorotov et al. (2020) (19)	Russia	394	Cardiac surgery	Children	RCT	Y	30 days	Dexamethasone	26.67	Post induction	High (7)
Mardani et al. (2012) (71)	Iran	110	CABG or valve	Adults	RCT	Unclear	Unclear	Dexamethasone	30	Pre and post surgery	Low (3)
Mayumi et al. (1997) (12)	Japan	24	Valve	Adults	RCT	Y	7 days	Methylprednisolone	200	Pre and post CPB	High (4)
McBride et al. (2004) (72)	Ireland	35	CABG	Adults	RCT	N	72 h	Methylprednisolone	150	Pre induction	Low (1)
Morariu et al. (2005) (73)	Netherlands	20	CABG	Adults	RCT	Y	Hospital stay	Dexamethasone	40	Pre induction and post surgery	High (5)
Morton et al. (1976) (74)	US	95	CABG	Adults	RCT	Y	30 days	Methylprednisolone	150	Pre induction	High (6)
Mott et al. (2001) (75)	US	246	Cardiac surgery	Children	RCT	Y	30 days	Methylprednisolone	25	Pre incision and post surgery	High (7)
Murphy et al. (2011) (76)	US	109	CABG or valve	Adults	RCT	Y	Hospital stay	Dexamethasone	6	During induction and initiation of CPB	High (7)
Niazi et al. (1979) (77)	US	90	CABG	Adults	RCT	Y	9 days	Methylprednisolone or dexamethasone	150/160	During sternotomy	High (4)
Oliver et al. (2004) (78)	US	125	CABG or valve	Adults	RCT	Y	ICU stay	Methylprednisolone	78	Pre induction and post surgery	High (4)
Prasongsukarn et al. (2005) (79)	Canada	86	CABG	Adults	RCT	Y	Hospital stay	Methylprednisolone and dexamethasone	78	Pre induction and post surgery	High (7)
Rao et al. (1977) (80)	US	150	CABG	Adults	RCT	Unclear	Hospital stay	Methylprednisolone	71	Pre CPB	Low (3)
Rubens et al. (2005) (81)	Canada	68	CABG	Adults	RCT	Y	Hospital stay	Methylprednisolone	71	Pre CPB	High (7)
Rumalla et al. (2001) (82)	US	13	CABG	Adults	RCT	N	6 months	Methylprednisolone	71	During induction	Low (1)
Sano et al. (2003) (83)	Japan	28	CABG or valve	Adults	RCT	N	7 days	Hydrocortisone	100	Pre and post CPB	Low (3)
Sano et al. (2006) (84)	Japan	60	CABG or valve	Adults	RCT	Y	Hospital stay	Hydrocortisone	100	Pre and post CPB	Low (3)
Schurr et al. (2001) (85)	Switzerland	50	CABG	Adults	RCT	N	Hospital stay	Methylprednisolone	50	Pre surgery	Low (3)
Sobieski et al. (2008) (86)	US	28	CABG	Adults	RCT	Y	72 h	Dexamethasone	38	Pre CPB	High (4)
Starobin et al. (2007) (87)	Israel	60	CABG	Adults	RCT	N	2 weeks	Betamethasone	3	Pre surgery	Low (2)
Suominen Jahnukainen et al. (2017/2018) (88, 89)	Finland	40	Cardiac surgery	Children	RCT	Y	Hospital stay	Methylprednisolone and hydrocortisone	25.6	Post induction and last for 5 days	High (7)
Taleska et al. (2020) (90)	Slovenia	76	CABG or valve or complex	Adults	RCT	Y	30 days	Methylprednisolone	71	During CPB	High (7)
Tassani et al. (1999) (91)	Germany	52	CABG	Adults	RCT	Y	Hospital stay	Methylprednisolone	71	Pre CPB	High (7)
Teoh et al. (1995) (92)	Canada	25	CABG	Adults	RCT	N	Hospital stay	Methylprednisolone	18	Pre induction	Low (3)
Toft et al. (1997) (93)	Denmark	16	Cardiac surgery	Adults	RCT	N	Hospital stay	Methylprednisolone	150	During induction	Low (3)
Toledo-Pereyra et al. (1980) (94)	US	95	Cardiac surgery	Children	RCT	Y	Hospital stay	Methylprednisolone	150	Pre CPB	Low (3)
Turkoz et al. (2001) (95)	Turkey	30	CABG	Adults	RCT	N	24 h	Methylprednisolone	150	Pre CPB	Low (3)
Vallejo et al. (1977) (96)	Spain	100	CABG	Adults	RCT	N	Hospital stay	Methylprednisolone	150	Pre CPB	Low (2)
Volk et al. (2001) (97)	Germany	39	CABG	Adults	RCT	Y	Hospital stay	Methylprednisolone	75	Pre CPB	High (4)
Volk et al. (2003) (98)	Germany	36	CABG	Adults	RCT	Y	Hospital stay	Methylprednisolone	75	Pre CPB	High (4)
Von Spiegel et al. (2001/2002) (99, 100)	Germany	20	CABG	Adults	RCT	Y	24 h	Dexamethasone	27	Post induction	High (5)
Vukovic et al. (2011) (101)	Serbia	57	CABG	Adults	RCT	Y	Hospital stay	Methylprednisolone	50	Post induction	High (4)
Wan et al. (1999) (102)	Belgium	20	CABG or valve	Adults	RCT	Y	Hospital stay	Methylprednisolone	150	During induction	High (4)
Weis et al. (2006) (103)	Switzerland	36	High risk CPB	Adults	RCT	Y	Hospital stay	Hydrocortisone	8	Pre induction and last for 3 days	High (5)
Weis et al. (2009) (104)	Germany	36	High risk CPB	Adults	RCT	Y	28 days	Hydrocortisone	8	Pre induction and last for 2 days	High (6)
Whitlock et al. (2006) (14)	Canada	60	CABG or valve or complex	Adults	RCT	Y	Hospital stay	Methylprednisolone	21	During induction and initiation of CPB	High (4)
Whitlock et al. (2015) (105)	Canada	7507	CABG or valve or complex	Adults	RCT	Y	6 months	Methylprednisolone	36	During induction and initiation of CPB	High (7)
Yared et al. (1998/2000) (106, 107)	US	236	CABG or valve	Adults	RCT	Y	Hospital stay	Dexamethasone	16	Post induction	Low (3)
Yared et al. (2007) (108)	US	71	CABG or valve	Adults	RCT	Y	Hospital stay	Dexamethasone	16	Post induction	High (4)
Yasser et al. (2009) (109)	Egypt	100	CABG	Adults	RCT	Unclear	Hospital stay	Dexamethasone	40	During induction and surgery	Low (3)
Yilmaz et al. (1999) (110)	Turkey	20	CABG	Adults	RCT	Y	Hospital stay	Methylprednisolone	5	During CPB	High (5)

*CABG, coronary artery bypass grafting. RCT, randomized controlled trial. CPB, cardiopulmonary bypass.*

*^a^* *Jadad score (18).*

**Table 2:** GRADE summary of findings.

Corticosteroids compared to placebo or saline for cardiopulmonary bypass						
Patient or population: cardiopulmonary bypass Intervention: corticosteroids Comparison: placebo or saline						
Outcomes	No. of participants (studies)	Anticipated absolute effects^a^ (95% CI)		Relative effect (95% CI)	Test for overall effect (p)	Certainty of the evidence (GRADE)
		Risk with placebo or saline	Risk with corticosteroids			
**Adult**						
Mortality	15780 (47 RCTs)	32 per 1,000	28 per 1,000 (23 to 33)	OR 0.86 (0.71 to 1.03)	1.66 (0.10)	⊕⊕⊕⊕ HIGH
≤20 mg/kg hydrocortisone	1312 (10 RCTs)	20 per 1,000	12 per 1,000 (5 to 26)	OR 0.57 (0.25 to 1.31)	1.32 (0.19)	⊕⊕⊕⊕ HIGH
20–40 mg/kg hydrocortisone	12317 (11 RCTs)	34 per 1,000	30 per 1,000 (25 to 37)	OR 0.87 (0.71 to 1.07)	1.32 (0.19)	⊕⊕⊕⊕ HIGH
40–100 mg/kg hydrocortisone	1025 (11 RCTs)	14 per 1,000	14 per 1,000 (5 to 36)	OR 0.99 (0.37 to 2.69)	0.01 (0.99)	⊕⊕⊕⊕ HIGH
>100 mg/kg hydrocortisone	1126 (15 RCTs)	40 per 1,000	35 per 1,000 (19 to 61)	OR 0.85 (0.47 to 1.55)	0.53 (0.60)	⊕⊕⊕⊕ HIGH
New atrial fibrillation	14745 (33 RCTs)	284 per 1,000	213 per 1,000 (185 to 246)	OR 0.68 (0.57 to 0.82)	4.15 (<0.0001)	⊕⊕⊕⊕ HIGH
≤20 mg/kg hydrocortisone	1279 (10 RCTs)	371 per 1,000	255 per 1,000 (206 to 309)	OR 0.58 (0.44 to 0.76)	3.90 (<0.0001)	⊕⊕⊕⊕ HIGH
20–40 mg/kg hydrocortisone	12394 (8 RCTs)	272 per 1,000	258 per 1,000 (243 to 274)	OR 0.93 (0.86 to 1.01)	1.83 (0.07)	⊕⊕⊕⊕ HIGH
40–100 mg/kg hydrocortisone	775 (9 RCTs)	351 per 1,000	286 per 1,000 (167 to 443)	OR 0.74 (0.37 to 1.47)	0.87 (0.39)	⊕⊕⊕⊕ HIGH
>100 mg/kg hydrocortisone	297 (6 RCTs)	264 per 1,000	225 per 1,000 (144 to 331)	OR 0.81 (0.47 to 1.38)	0.78 (0.43)	⊕⊕⊕⊕ HIGH
Myocardial infarction	14669 (25 RCTs)	65 per 1,000	77 per 1,000 (68 to 86)	OR 1.19 (1.05 to 1.35)	2.66 (0.008)	⊕⊕⊕⊕ HIGH
≤20 mg/kg hydrocortisone	1115 (6 RCTs)	20 per 1,000	19 per 1,000 (9 to 42)	OR 0.96 (0.43 to 2.17)	0.09 (0.93)	⊕⊕⊕⊕ HIGH
20–40 mg/kg hydrocortisone	12242 (5 RCTs)	72 per 1,000	86 per 1,000 (76 to 97)	OR 1.21 (1.06 to 1.38)	2.79 (0.005)	⊕⊕⊕⊕ HIGH
40–100 mg/kg hydrocortisone	780 (6 RCTs)	33 per 1,000	28 per 1,000 (13 to 61)	OR 0.85 (0.38 to 1.88)	0.41 (0.68)	⊕⊕⊕⊕ HIGH
>100 mg/kg hydrocortisone	532 (8 RCTs)	49 per 1,000	56 per 1,000 (28 to 111)	OR 1.15 (0.55 to 2.43)	0.37 (0.71)	⊕⊕⊕⊕ HIGH
Pulmonary complications	8932 (17 RCTs)	92 per 1,000	85 per 1,000 (73 to 96)	OR 0.91 (0.78 to 1.05)	1.30 (0.20)	⊕⊕⊕⊕ HIGH
≤20 mg/kg hydrocortisone	822 (8 RCTs)	42 per 1,000	41 per 1,000 (21 to 78)	OR 0.97 (0.48 to 1.93)	0.10 (0.92)	⊕⊕⊕⊕ HIGH
20–40 mg/kg hydrocortisone	7567 (2 RCTs)	99 per 1,000	91 per 1,000 (79 to 105)	OR 0.91 (0.78 to 1.06)	1.22 (0.22)	⊕⊕⊕⊕ HIGH
40–100 mg/kg hydrocortisone	433 (5 RCTs)	65 per 1,000	56 per 1,000 (27 to 115)	OR 0.86 (0.40 to 1.88)	0.37 (0.71)	⊕⊕⊕⊕ HIGH
>100 mg/kg hydrocortisone	110 (2 RCTs)	73 per 1,000	56 per 1,000 (14 to 203)	OR 0.76 (0.18 to 3.24)	0.37 (0.71)	⊕⊕⊕⊕ HIGH
Kidney injury	12826 (13 RCTs)	34 per 1,000	28 per 1,000 (23 to 34)	OR 0.83 (0.68 to 1.01)	1.88 (0.06)	⊕⊕⊕⊕ HIGH
≤20 mg/kg hydrocortisone	520 (6 RCTs)	43 per 1,000	17 per 1,000 (6 to 48)	OR 0.38 (0.13 to 1.11)	1.77 (0.08)	⊕⊕⊕⊕ HIGH
20–40 mg/kg hydrocortisone	12142 (4 RCTs)	33 per 1,000	28 per 1,000 (23 to 34)	OR 0.84 (0.68 to 1.03)	1.66 (0.10)	⊕⊕⊕⊕ HIGH
40–100 mg/kg hydrocortisone	164 (2 RCTs)	49 per 1,000	72 per 1,000 (20 to 225)	OR 1.49 (0.40 to 5.58)	0.59 (0.55)	⊕⊕⊕⊕ HIGH
>100 mg/kg hydrocortisone	(1 RCT)	0 per 1,000	0 per 1,000 (0 to 0)	not estimable	–	⊕⊕⊕⊕ HIGH
Postoperative infection	14880 (37 RCTs)	81 per 1,000	77 per 1,000 (69 to 86)	OR 0.95 (0.84 to 1.07)	0.84 (0.40)	⊕⊕⊕⊕ HIGH
≤20 mg/kg hydrocortisone	1299 (11 RCTs)	67 per 1,000	57 per 1,000 (36 to 87)	OR 0.84 (0.52 to 1.33)	0.75 (0.45)	⊕⊕⊕⊕ HIGH
20–40 mg/kg hydrocortisone	12295 (7 RCTs)	86 per 1,000	82 per 1,000 (73 to 93)	OR 0.95 (0.84 to 1.08)	0.76 (0.45)	⊕⊕⊕⊕ HIGH
40–100 mg/kg hydrocortisone	612 (10 RCTs)	56 per 1,000	59 per 1,000 (31 to 109)	OR 1.06 (0.54 to 2.09)	0.17 (0.87)	⊕⊕⊕⊕ HIGH
>100 mg/kg hydrocortisone	674 (9 RCTs)	39 per 1,000	41 per 1,000 (20 to 83)	OR 1.07 (0.51 to 2.26)	0.18 (0.86)	⊕⊕⊕⊕ HIGH
Neurological complications (strok)	13439 (18 RCTs)	21 per 1,000	18 per 1,000 (14 to 22)	OR 0.85 (0.66 to 1.08)	1.33 (0.18)	⊕⊕⊕⊕ HIGH
≤20 mg/kg hydrocortisone	626 (4 RCTs)	19 per 1,000	16 per 1,000 (5 to 53)	OR 0.85 (0.25 to 2.82)	0.27 (0.79)	⊕⊕⊕⊕ HIGH
20–40 mg/kg hydrocortisone	12142 (4 RCTs)	19 per 1,000	17 per 1,000 (13 to 22)	OR 0.89 (0.68 to 1.16)	0.86 (0.39)	⊕⊕⊕⊕ HIGH
40–100 mg/kg hydrocortisone	454 (6 RCTs)	52 per 1,000	26 per 1,000 (10 to 64)	OR 0.48 (0.18 to 1.24)	1.52 (0.13)	⊕⊕⊕⊕ HIGH
>100 mg/kg hydrocortisone	217 (4 RCTs)	56 per 1,000	47 per 1,000 (15 to 135)	OR 0.83 (0.26 to 2.66)	0.31 (0.76)	⊕⊕⊕⊕ HIGH
Gastro-intestinal bleeding	12535 (6 RCTs)	10 per 1,000	12 per 1,000 (9 to 17)	OR 1.22 (0.88 to 1.69)	1.17 (0.24)	⊕⊕⊕⊕ HIGH
Mechanical ventilation time (hours)	7005 (42 RCTs)	The mean mechanical ventilation time (hours) was 8.39	MD 0.48 lower (1.04 lower to 0.09 higher)	–	1.66 (0.10)	⊕⊕⊕⊕ HIGH
≤20 mg/kg hydrocortisone	1119 (11 RCTs)	The mean mechanical ventilation time (hours) was 9.82	MD 2.74 lower (4.14 lower to 1.33 lower)	–	3.82 (0.0001)	⊕⊕⊕⊕ HIGH
20–40 mg/kg hydrocortisone	4946 (12 RCTs)	The mean mechanical ventilation time (hours) was 7.44	MD 0.53 lower (1.39 lower to 0.34 higher)	–	1.20 (0.23)	⊕⊕⊕⊕ HIGH
40–100 mg/kg hydrocortisone	639 (12 RCTs)	The mean mechanical ventilation time (hours) was 12.15	MD 0.94 lower (2.44 lower to 0.56 higher)	–	1.23 (0.22)	⊕⊕⊕⊕ HIGH
>100 mg/kg hydrocortisone	301 (7 RCTs)	The mean mechanical ventilation time (hours) was 10.91	MD 3.82 higher (0.76 higher to 6.87 higher)	–	2.45 (0.01)	⊕⊕⊕⊕ HIGH
Hyperglycemia requiring insulin infusion	8316 (14 RCTs)	113 per 1,000	196 per 1,000 (131 to 284)	OR 1.91 (1.18 to 3.11)	2.61 (0.009)	⊕⊕⊕⊕ HIGH
≤20 mg/kg hydrocortisone	421 (4 RCTs)	233 per 1,000	343 per 1,000 (201 to 518)	OR 1.72 (0.83 to 3.55)	1.46 (0.15)	⊕⊕⊕⊕ HIGH
20–40 mg/kg hydrocortisone	7547 (3 RCTs)	103 per 1,000	335 per 1,000 (64 to 786)	OR 4.41 (0.60 to 32.10)	1.46 (0.14)	⊕⊕⊕⊕ HIGH
40–100 mg/kg hydrocortisone	120 (2 RCTs)	417 per 1,000	895 per 1,000 (502 to 986)	OR 11.88 (1.41 to 100.00)	2.28 (0.02)	⊕⊕⊕⊕ HIGH
>100 mg/kg hydrocortisone	228 (5 RCTs)	88 per 1,000	129 per 1,000 (59 to 258)	OR 1.53 (0.65 to 3.59)	0.98 (0.33)	⊕⊕⊕⊕ HIGH
Delirium	12181 (6 RCTs)	92 per 1,000	83 per 1,000 (74 to 93)	OR 0.89 (0.79 to 1.01)	1.78 (0.08)	⊕⊕⊕⊕ HIGH
LOS in ICU (days)	14068 (42 RCTs)	The mean LOS ICU (days) was 1.69	MD 0.27 lower (0.34 lower to 0.19 lower)	–	6.66 (<0.00001)	⊕⊕⊕⊕ HIGH
≤20 mg/kg hydrocortisone	641 (9 RCTs)	The mean LOS ICU (days) was 2.55	MD 1.48 lower (2.73 lower to 0.22 lower)	–	2.31 (0.02)	⊕⊕⊕⊕ HIGH
20–40 mg/kg hydrocortisone	12454 (13 RCTs)	The mean LOS ICU (days) was 1.60	MD 0.15 lower (0.23 lower to 0.07 lower)	–	3.83 (0.0001)	⊕⊕⊕⊕ HIGH
40–100 mg/kg hydrocortisone	743 (15 RCTs)	The mean LOS ICU (days) was 1.76	MD 0.19 lower (0.42 lower to 0.03 higher)	–	1.67 (0.10)	⊕⊕⊕⊕ HIGH
>100 mg/kg hydrocortisone	230 (5 RCTs)	The mean LOS ICU (days) was 3.93	MD 0.08 lower (0.31 lower to 0.15 higher)	–	0.69 (0.49)	⊕⊕⊕⊕ HIGH
LOS in hospital (days)	13806 (32 RCTs)	The mean LOS hospital (days) was 9.07	MD 0.66 lower (1.03 lower to 0.3 lower)	–	3.59 (0.0003)	⊕⊕⊕⊕ HIGH
≤20 mg/kg hydrocortisone	395 (7 RCTs)	The mean LOS hospital (days) was 11.39	MD 2.29 lower (4.51 lower to 0.07 lower)	–	2.02 (0.04)	⊕⊕⊕⊕ HIGH
20–40 mg/kg hydrocortisone	12306 (7 RCTs)	The mean LOS hospital (days) was 9.09	MD 0.16 lower (0.72 lower to 0.41 higher)	–	0.54 (0.59)	⊕⊕⊕⊕ HIGH
40–100 mg/kg hydrocortisone	490 (10 RCTs)	The mean LOS hospital (days) was 9.24	MD 0.19 higher (0.52 lower to 0.9 higher)	–	0.54 (0.59)	⊕⊕⊕⊕ HIGH
>100 mg/kg hydrocortisone	615 (8 RCTs)	The mean LOS hospital (days) was 7.27	MD 0.91 lower (1.63 lower to 0.2 lower)	–	2.49 (0.01)	⊕⊕⊕⊕ HIGH
Duration of CPB (minutes)	15457 (56 RCTs)	The mean duration of CPB (minutes) was 111.02	MD 0.94 higher (0.69 lower to 2.56 higher)	–	1.13 (0.26)	⊕⊕⊕⊕ HIGH
≤20 mg/kg hydrocortisone	1445 (14 RCTs)	The mean duration of CPB (minutes) was 86.96	MD 0.47 higher (1.84 lower to 2.77 higher)	–	0.40 (0.69)	⊕⊕⊕⊕ HIGH
20–40 mg/kg hydrocortisone	12432 (13 RCTs)	The mean duration of CPB (minutes) was 114.87	MD 0.89 higher (3.36 lower to 5.15 higher)	–	0.41 (0.68)	⊕⊕⊕⊕ HIGH
40–100 mg/kg hydrocortisone	599 (12 RCTs)	The mean duration of CPB (minutes) was 96.62	MD 2.97 higher (1.38 lower to 7.31 higher)	–	1.34 (0.18)	⊕⊕⊕⊕ HIGH
>100 mg/kg hydrocortisone	981 (17 RCTs)	The mean duration of CPB (minutes) was 105.74	MD 0.11 lower (2.79 lower to 2.58 higher)	–	0.08 (0.94)	⊕⊕⊕⊕ HIGH
Duration of procedure (minutes)	5476 (16 RCTs)	The mean duration of procedure (minutes) was 238.33	MD 11.93 higher (2.28 higher to 21.58 higher)	–	2.42 (0.02)	⊕⊕⊕⊕ HIGH
≤20 mg/kg hydrocortisone	538 (3 RCTs)	The mean duration of procedure (minutes) was 199.30	MD 3.73 higher (1.72 lower to 9.19 higher)	–	1.34 (0.18)	⊕⊕⊕⊕ HIGH
20–40 mg/kg hydrocortisone	4519 (3 RCTs)	The mean duration of procedure (minutes) was 241.88	MD 9.64 higher (8.92 lower to 28.2 higher)	–	1.02 (0.31)	⊕⊕⊕⊕ HIGH
40–100 mg/kg hydrocortisone	219 (4 RCTs)	The mean duration of procedure (minutes) was 214.37	MD 11.78 higher (10.18 lower to 33.74 higher)	–	1.05 (0.29)	⊕⊕⊕⊕ HIGH
>100 mg/kg hydrocortisone	200 (6 RCTs)	The mean duration of procedure (minutes) was 289.88	MD 14.03 higher (10.19 lower to 38.25 higher)	–	1.14 (0.26)	⊕⊕⊕⊕ HIGH
Postoperative bleeding (mL)	1084 (13 RCTs)	The mean postoperative bleeding (mL) was 763.32	MD 99.73 lower (169.45 lower to 30 lower)	–	2.80 (0.005)	⊕⊕⊕⊕ HIGH
≤20 mg/kg hydrocortisone	569 (3 RCTs)	The mean postoperative bleeding (mL) was 666.99	MD 20.83 lower (56.43 lower to 14.78 higher)	–	1.15 (0.25)	⊕⊕⊕⊕ HIGH
20–40 mg/kg hydrocortisone	100 (3 RCTs)	The mean postoperative bleeding (mL) was 847.00	MD 66.96 lower (192.33 lower to 58.41 higher)	–	1.05 (0.30)	⊕⊕⊕⊕ HIGH
40–100 mg/kg hydrocortisone	295 (4 RCTs)	The mean postoperative bleeding (mL) was 919.53	MD 194.85 lower (302.08 lower to 87.61 lower)	–	3.56 (0.0004)	⊕⊕⊕⊕ HIGH
>100 mg/kg hydrocortisone	120 (3 RCTs)	The mean postoperative bleeding (mL) was 758.50	MD 83.82 lower (130.62 lower to 37.03 lower)	–	3.51 (0.0004)	⊕⊕⊕⊕ HIGH
Vaso-active medication use	1405 (20 RCTs)	272 per 1,000	274 per 1,000 (205 to 355)	OR 1.01 (0.69 to 1.47)	0.03 (0.98)	⊕⊕⊕⊕ HIGH
≤20 mg/kg hydrocortisone	639 (4 RCTs)	177 per 1,000	164 per 1,000 (111 to 238)	OR 0.91 (0.58 to 1.45)	0.38 (0.70)	⊕⊕⊕⊕ HIGH
20–40 mg/kg hydrocortisone	183 (6 RCTs)	462 per 1,000	269 per 1,000 (93 to 564)	OR 0.43 (0.12 to 1.51)	1.32 (0.19)	⊕⊕⊕⊕ HIGH
40–100 mg/kg hydrocortisone	80 (2 RCTs)	293 per 1,000	303 per 1,000 (87 to 666)	OR 1.05 (0.23 to 4.81)	0.06 (0.95)	⊕⊕⊕⊕ HIGH
>100 mg/kg hydrocortisone	503 (8 RCTs)	320 per 1,000	414 per 1,000 (311 to 525)	OR 1.50 (0.96 to 2.35)	1.78 (0.08)	⊕⊕⊕⊕ HIGH
IL-6 concentrations at 24 h (pg/mL)	506 (14 RCTs)	The mean IL-6 concentrations at 24 h (pg/mL) was 310.89	MD 139.77 lower (161.56 lower to 117.97 lower)	–	12.57 (<0.00001)	⊕⊕⊕⊕ HIGH
CRP concentrations at 24 h (µg/mL)	631 (4 RCTs)	The mean CRP concentrations at 24 h (µg/mL) was 139.04	MD 8.98 lower (20.41 lower to 2.45 higher)	–	1.54 (0.12)	⊕⊕⊕⊕ HIGH
TNF-α concentrations at 24 h (pg/mL)	199 (6 RCTs)	The mean tNF-α concentrations at 24 h (pg/mL) was 16.73	MD 4.23 lower (6.85 lower to 1.6 lower)	–	3.16 (0.002)	⊕⊕⊕⊕ HIGH
IL-8 concentrations at 24 h (pg/mL)	199 (6 RCTs)	The mean IL-8 concentrations at 24 h (pg/mL) was 15.01	MD 5.81 lower (10.96 lower to 0.66 lower)	–	2.21 (0.03)	⊕⊕⊕⊕ HIGH
IL-10 concentrations at 24 h (pg/mL)	109 (3 RCTs)	The mean IL-10 concentrations at 24 h (pg/mL) was 14.34	MD 6.13 higher (2.93 lower to 15.19 higher)	–	1.33 (0.19)	⊕⊕⊕⊕ HIGH
Re-intubation	258 (5 RCTs)	102 per 1,000	38 per 1,000 (14 to 97)	OR 0.35 (0.13 to 0.95)	2.06 (0.04)	⊕⊕⊕⊕ HIGH
Re-thoracotomy	935 (9 RCTs)	27 per 1,000	32 per 1,000 (16 to 64)	OR 1.17 (0.57 to 2.40)	0.44 (0.66)	⊕⊕⊕⊕ HIGH
**Pediatric**						
Mortality	931 (12 RCTs)	48 per 1,000	28 per 1,000 (15 to 53)	OR 0.57 (0.30 to 1.11)	1.64 (0.10)	⊕⊕⊕⊕ HIGH
≤50 mg/kg hydrocortisone	531 (6 RCTs)	22 per 1,000	16 per 1,000 (5 to 48)	OR 0.71 (0.23 to 2.19)	0.59 (0.55)	⊕⊕⊕⊕ HIGH
>50 mg/kg hydrocortisone	400 (6 RCTs)	82 per 1,000	44 per 1,000 (20 to 95)	OR 0.51 (0.23 to 1.17)	1.59 (0.11)	⊕⊕⊕⊕ HIGH
Kidney injury	659 (5 RCTs)	236 per 1,000	127 per 1,000 (64 to 238)	OR 0.47 (0.22 to 1.01)	1.94 (0.05)	⊕⊕⊕⊕ HIGH
≤50 mg/kg hydrocortisone	483 (4 RCTs)	127 per 1,000	40 per 1,000 (13 to 123)	OR 0.29 (0.09 to 0.96)	2.02 (0.04)	⊕⊕⊕⊕ HIGH
>50 mg/kg hydrocortisone	176 (1 RCT)	516 per 1,000	457 per 1,000 (319 to 604)	OR 0.79 (0.44 to 1.43)	0.78 (0.44)	⊕⊕⊕⊕ HIGH
ECMO	570 (2 RCTs)	47 per 1,000	19 per 1,000 (6 to 52)	OR 0.38 (0.13 to 1.10)	1.79 (0.07)	⊕⊕⊕⊕ HIGH
Postoperative infection	304 (5 RCTs)	70 per 1,000	48 per 1,000 (18 to 122)	OR 0.68 (0.25 to 1.85)	0.75 (0.45)	⊕⊕⊕⊕ HIGH
Hyperglycemia requiring insulin infusion	256 (3 RCTs)	67 per 1,000	208 per 1,000 (99 to 387)	OR 3.68 (1.53 to 8.84)	2.91 (0.004)	⊕⊕⊕⊕ HIGH
Mechanical ventilation time (hours)	505 (8 RCTs)	The mean mechanical ventilation time (hours) was 80.35	MD 7.37 lower (15.53 lower to 0.79 higher)	–	1.77 (0.08)	⊕⊕⊕⊕ HIGH
≤50 mg/kg hydrocortisone	100 (3 RCTs)	The mean mechanical ventilation time (hours) was 103.04	MD 28.23 lower (73.47 lower to 17.01 higher)	–	1.22 (0.22)	⊕⊕⊕⊕ HIGH
>50 mg/kg hydrocortisone	405 (5 RCTs)	The mean mechanical ventilation time (hours) was 74.94	MD 7.47 lower (19.89 lower to 4.95 higher)	–	1.18 (0.24)	⊕⊕⊕⊕ HIGH
Duration of CPB (minutes)	875 (14 RCTs)	The mean duration of CPB (minutes) was 132.73	MD 11.54 lower (14.32 lower to 8.75 lower)	–	8.12 (<0.00001)	⊕⊕⊕⊕ HIGH
≤50 mg/kg hydrocortisone	441 (8 RCTs)	The mean duration of CPB (minutes) was 129.29	MD 12.06 lower (15.19 lower to 8.94 lower)	–	7.56 (<0.00001)	⊕⊕⊕⊕ HIGH
>50 mg/kg hydrocortisone	434 (6 RCTs)	The mean duration of CPB (minutes) was 136.04	MD 9.52 lower (15.63 lower to 3.41 lower)	–	3.05 (0.02)	⊕⊕⊕⊕ HIGH
LOS ICU (days)	405 (8 RCTs)	The mean LOS ICU (days) was 8.08	MD 0 (0.12 lower to 0.11 higher)	–	0.05 (0.96)	⊕⊕⊕⊕ HIGH
≤50 mg/kg hydrocortisone	100 (3 RCTs)	The mean LOS ICU (days) was 7.52	MD 1.06 lower (3.08 lower to 0.96 higher)	–	1.03 (0.30)	⊕⊕⊕⊕ HIGH
>50 mg/kg hydrocortisone	305 (5 RCTs)	The mean LOS ICU (days) was 8.25	MD 0.03 higher (0.34 lower to 0.39 higher)	–	0.14 (0.89)	⊕⊕⊕⊕ HIGH
Inotropic score	454 (7 RCTs)	The mean inotropic score was 12.88	MD 0.09 lower (0.39 lower to 0.22 higher)	–	0.56 (0.58)	⊕⊕⊕⊕ HIGH
Highest/24 h temperature (°C)	216 (7 RCTs)	The mean highest/24 h temperature (°C) was 37.63	MD 0.07 lower (0.43 lower to 0.29 higher)	–	0.40 (0.69)	⊕⊕⊕⊕ HIGH
≤50 mg/kg hydrocortisone	87 (3 RCTs)	The mean highest/24 h temperature (°C) was 37.38	MD 0.28 higher (0.08 lower to 0.63 higher)	–	1.54 (0.12)	⊕⊕⊕⊕ HIGH
>50 mg/kg hydrocortisone	129 (4 RCTs)	The mean highest/24 h temperature (°C) was 37.80	MD 0.26 lower (0.61 lower to 0.1 higher)	–	1.42 (0.16)	⊕⊕⊕⊕ HIGH
Highest/24 h glucose concentrations (mg/dl)	236 (3 RCTs)	The mean highest/24 h glucose concentrations (mg/dl) was 138.50	MD 17.94 higher (0.17 lower to 36.06 higher)	–	1.94 (0.05)	⊕⊕⊕⊕ HIGH
Highest/24 h lactate concentrations (mmol/l)	305 (5 RCTs)	The mean highest/24 h lactate concentrations (mmol/l) was 3.04	MD 0.06 lower (0.17 lower to 0.05 higher)	–	1.07 (0.28)	⊕⊕⊕⊕ HIGH
Highest/24 h CRP concentrations (µg/mL) (≤50 mg/kg hydrocortisone)	98 (3 RCTs)	The mean highest/24 h CRP concentrations (µg/mL) was 50.58	MD 20.12 lower (28.68 lower to 11.55 lower)	–	4.60 (<0.00001)	⊕⊕⊕⊕ HIGH
Highest/24 h IL-6 concentrations (pg/mL)	316 (8 RCTs)	The mean highest/24 h IL-6 concentrations (pg/mL) was 211.77	MD 108.6 lower (206.02 lower to 11.18 lower)	–	2.18 (0.03)	⊕⊕⊕⊕ HIGH
≤50 mg/kg hydrocortisone	98 (4 RCTs)	The mean highest/24 h IL-6 concentrations (pg/mL) was 386.84	MD 102.67 lower (185.42 lower to 19.93 lower)	–	2.43 (0.02)	⊕⊕⊕⊕ HIGH
>50 mg/kg hydrocortisone	218 (4 RCTs)	The mean highest/24 h IL-6 concentrations (pg/mL) was 133.07	MD 85.72 lower (308.08 lower to 136.64 higher)	–	0.76 (0.45)	⊕⊕⊕⊕ HIGH
Highest/24 h IL-10 concentrations (pg/mL)	258 (5 RCTs)	The mean highest/24 h IL-10 concentrations (pg/mL) was 298.04	MD 227.35 higher (169.67 higher to 285.03 higher)	–	7.73 (<0.00001)	⊕⊕⊕⊕ HIGH
≤50 mg/kg hydrocortisone	40 (1 RCT)	The mean highest/24 h IL-10 concentrations (pg/mL) was 48.1	MD 321.1 higher (99.29 higher to 542.91 higher)	–	2.84 (0.005)	⊕⊕⊕⊕ HIGH
>50 mg/kg hydrocortisone	218 (4 RCTs)	The mean highest/24 h IL-10 concentrations (pg/mL) was 343.91	MD 220.55 higher (160.82 higher to 280.28 higher)	–	7.24 (<0.00001)	⊕⊕⊕⊕ HIGH

*CI, Confidence interval; OR, odds ratio; MD, mean difference.*

***GRADE Working Group grades of evidence***

***High certainty:*** *We are very confident that the true effect lies close to that of the estimate of the effect.*

***Moderate certainty:*** *We are moderately confident in the effect estimate: The true effect is likely to be close to the estimate of the effect, but there is a possibility that it is substantially different.*

***Low certainty:*** *Our confidence in the effect estimate is limited: The true effect may be substantially different from the estimate of the effect.*

***Very low certainty:*** *We have very little confidence in the effect estimate: The true effect is likely to be substantially different from the estimate of effect.*

^a^ ***The risk in the intervention group*** *(and its 95% confidence interval) is based on the assumed risk in the comparison group and the **relative effect** of the intervention (and its 95% CI).*

**Table 3:** Summary of corticosteroids impact on adults and pediatric.

Group	Outcome	OR	CI	*p*	*I* ^2^	Impact
Adult	Myocardial infarction	1.19	1.05–1.35	0.008	0%	Increased
	Insulin infusion	1.91	1.18–3.11	0.009	46%	Increased
	Mortality	0.86	0.71–1.03	0.1	0%	Unaffected
	Postoperative atrial fibrillation	0.68	0.57–0.82	<0.0001	48%	Reduced
	ICU stay	−0.27	−0.34, −0.19	<0.00001	93%	Reduced
	Hospital stay	–0.66	−1.03, −0.30	0.0003	95%	Reduced
	Postoperative bleeding	−99.73	−169.45, −30.00	0.005	84%	Reduced
	Re-intubation	0.35	0.13–0.95	0.04	6%	Reduced
	IL-6	−139.77	−161.56, −117.97	<0.001	99%	Reduced
	TNF-α	−4.23	−6.85, −1.60	0.002	88%	Reduced
	IL-8	−5.81	−10.96, −0.66	0.003	97%	Reduced
	Kidney injury	0.83	0.68–1.01	0.06	0%	Unaffected
	Pulmonary complications	0.91	0.78–1.05	0.2	0%	Unaffected
	Stroke	0.85	0.66–1.08	0.18	0%	Unaffected
	Gastrointestinal bleeding	1.22	0.88–1.69	0.24	0%	Unaffected
	Postoperative infection	0.95	0.84–1.07	0.4	0%	Unaffected
	Delirium	0.89	0.79–1.01	0.08	45%	Unaffected
	Mechanical ventilation time	−0.48	−1.04, 0.09	0.1	94%	Unaffected
Adult ≤20 mg/kg hydrocortisone	Myocardial infarction	0.96	0.43–2.17	0.93	0%	Unaffected
	Insulin infusion	1.72	0.83–3.55	0.15	36%	Unaffected
	Mortality	0.57	0.25–1.31	0.19	0%	Unaffected
	Postoperative atrial fibrillation	0.58	0.44–0.76	<0.0001	13%	Reduced
	ICU stay	−1.48	−2.73,−0.22	<0.00001	96%	Reduced
	Hospital stay	−2.29	−4.51,−0.07	<0.00001	96%	Reduced
	Mechanical ventilation time	−2.74	−4.14,−1.33	0.0001	92%	Reduced
	Postoperative bleeding	−20.83	−56.43,14.78	0.25	0%	Unaffected
	Kidney injury	0.38	0.13–1.11	0.08	0%	Unaffected
	Pulmonary complications	0.97	0.48–1.93	0.92	0%	Unaffected
	Stroke	0.85	0.25–2.82	0.79	0%	Unaffected
	Postoperative infection	0.84	0.52–1.33	0.45	0%	Unaffected
Pediatric	Reduced CRP	−20.12	−28.68, −11.55	<0.001	42%	Reduced
	Reduced IL-6	−108.60	−206.02, −11.18	0.03	95%	Reduced
	Increased IL-10	227.35	169.67–285.03	<0.001	40%	Increased
	Decreased CPB time	−11.54	−14.32, −8.75	<0.001	5%	Reduced
	Increased insulin infusion	3.68	1.53–8.84	0.004	48%	Increased
	mortality	0.57	0.30–1.11	0.1	0%	Unaffected
	kidney injury	0.47	0.22–1.01	0.05	46%	Unaffected
	ECMO use	0.38	0.13–1.10	0.07	0%	Unaffected
	postoperative infection	0.68	0.25–1.85	0.45	0%	Unaffected
	mechanical ventilation time	−7.37	−15.53, 0.79	0.08	83%	Unaffected
	ICU length of stay	−0.00	−0.12, 0.11	0.96	0%	Unaffected
Pediatric ≤50 mg/kg hydrocortisone	Reduced IL-6	−102.67	−185.42,−19.93	0.02	78%	Reduced
	Decreased CPB time	−12.06	−15.19,−8.94	<0.001	3%	Reduced
	kidney injury	0.29	0.09–0.96	0.04	49%	Reduced
	mortality	0.71	0.23–2.19	0.55	0%	Unaffected
	mechanical ventilation time	−28.23	−73.47,17.01	0.22	65%	Unaffected
	ICU length of stay	−1.06	−3.08,0.96	0.3	51%	Unaffected

**Figure 2:**
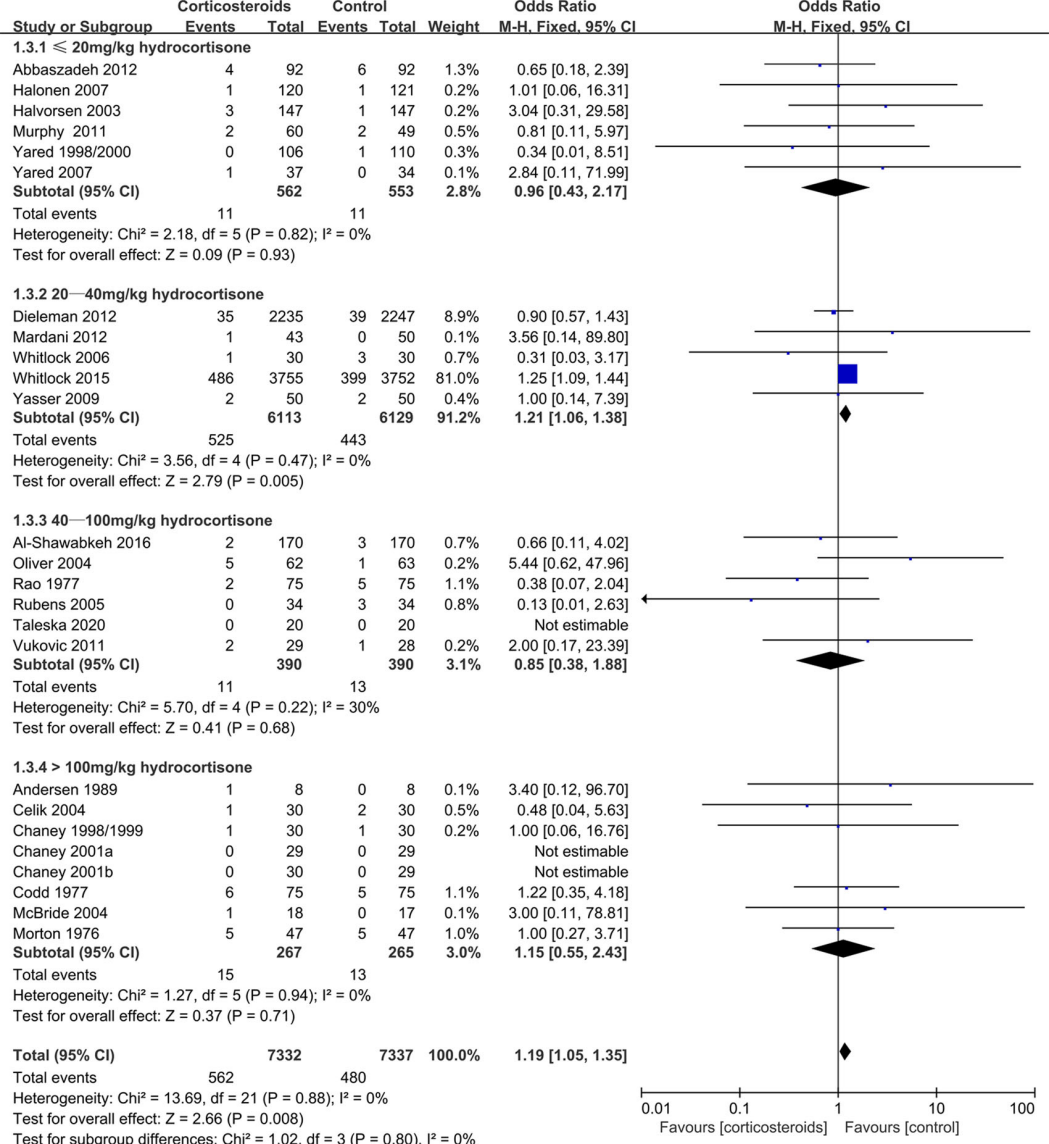
Impact of corticosteroids on myocardial infarction (adult).

**Figure 3:**
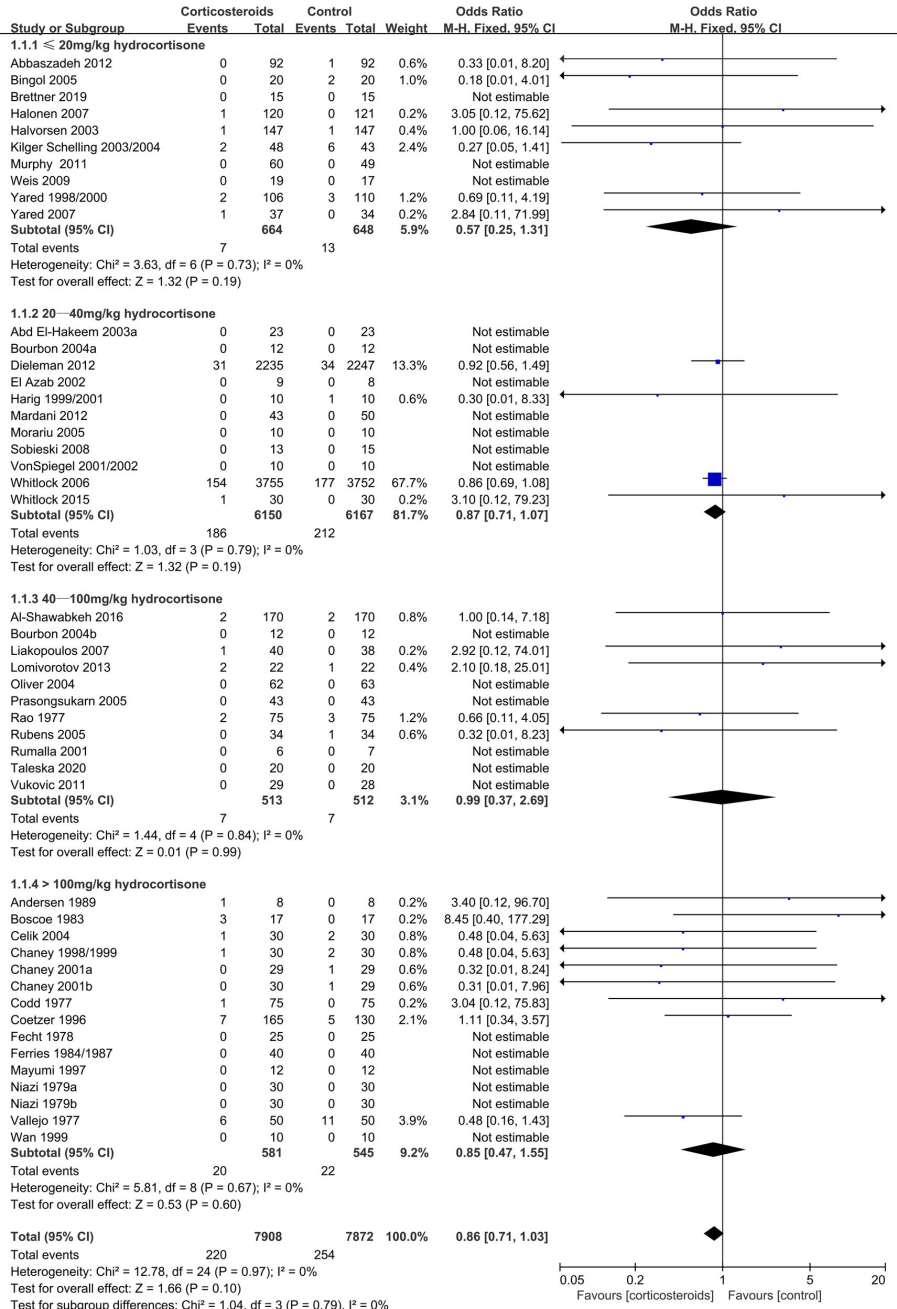
Impact of corticosteroids on mortality (adult).

During adult cardiac surgery with CPB, corticosteroids prophylaxis was associated with increased risks of myocardial infarction (OR: 1.19, 95% CI: 1.05–1.35; *p* = 0.008, *I*^2 ^= 0%) (Figure 2) and insulin infusion (OR: 1.91, 95% CI: 1.18–3.11; *p* = 0.009, *I*^2 ^= 46%) (Supplementary Figure S1), with no obvious improvement in mortality (OR: 0.86, 95% CI: 0.71–1.03; *p* = 0.10, *I*^2 ^= 0%) (Figure 3).

**Figure 4:**
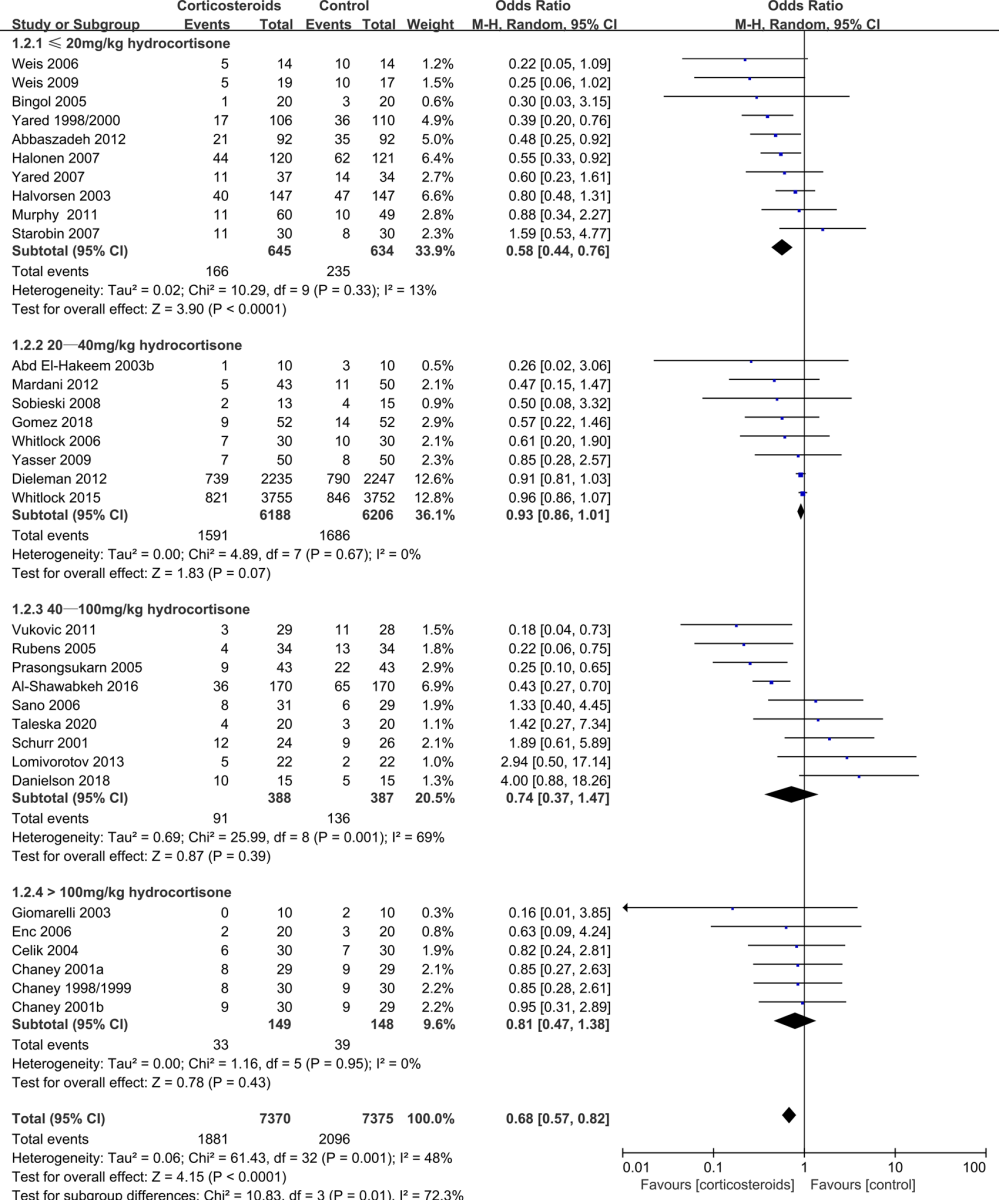
Impact of corticosteroids on postoperative new atrial fibrillation (adult).

However, corticosteroids prophylaxis reduced the risk of postoperative atrial fibrillation (OR: 0.68, 95% CI: 0.57–0.82; *p* < 0.0001, *I*^2 ^= 48%) (Figure 4), shortened the ICU stay (MD: −0.27 days, 95% CI: −0.34, −0.19 days; *p* < 0.001, *I*^2 ^= 93%), and shortened the hospital stay (MD: −0.66 days, 95% CI: −1.03, −0.30 days; *p* = 0.0003, *I*^2^ = 95%) (Supplementary Figures S2, S3). In addition, corticosteroids prophylaxis was associated with reduced postoperative bleeding and a reduced risk of re-intubation (Table 3 and Supplementary Figures S4, S5).

Corticosteroids prophylaxis also reduced the blood concentrations of some inflammatory markers in adult patients, which included IL-6, TNF-α, and IL-8 (Table 3 and Supplementary Figures S6–S8). Among children, corticosteroids prophylaxis was associated with a significantly lower peak CRP concentration, a significantly lower IL-6 concentration, and a significantly higher IL-10 concentration (Table 3 and Supplementary Figures S9–S11).

**Figure 5:**
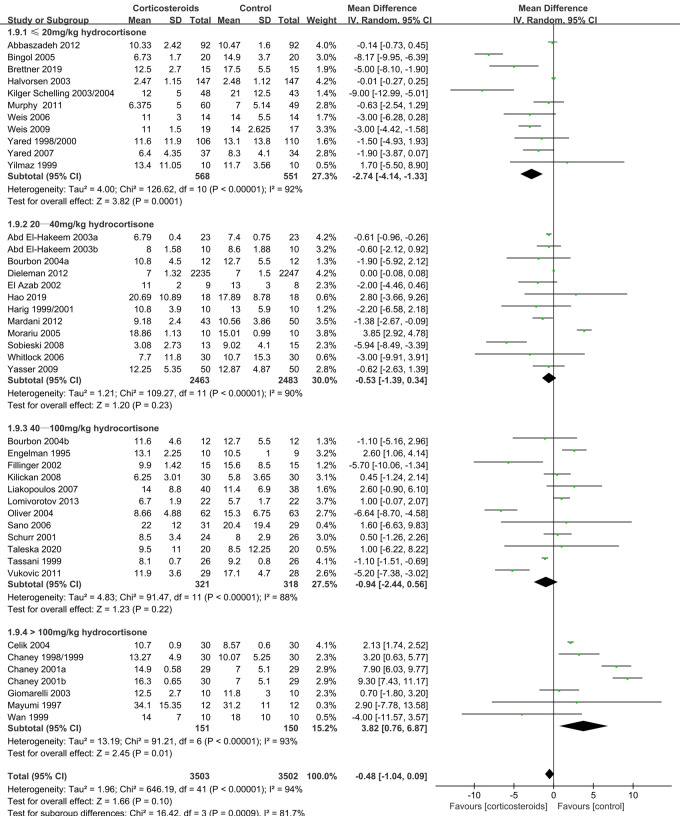
Impact of corticosteroids on mechanical ventilation time (hours) (adult).

Relative to the placebo group, corticosteroids prophylaxis was not associated with significant improvements in terms of kidney injury, pulmonary complications, stroke, gastrointestinal bleeding, postoperative infection or mechanical ventilation time (Table 3 and Figure 5, Supplementary Figures S12–S17).

**Figure 6:**
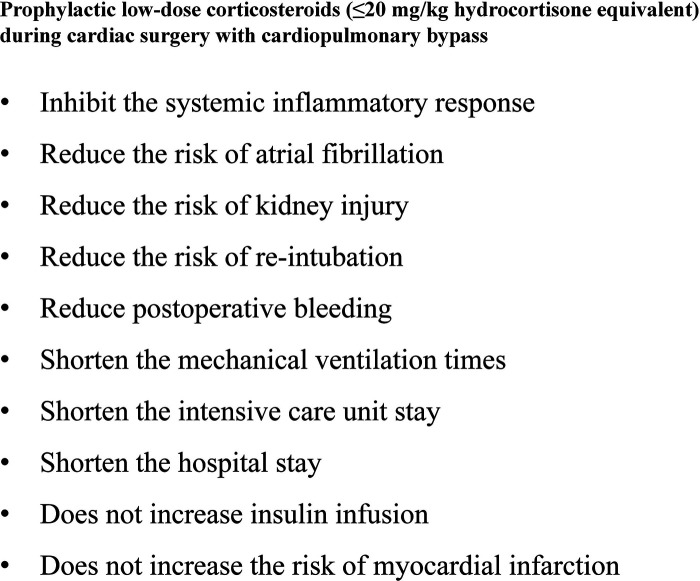
Highlight of the impact of low-dose corticosteroids.

Subgroup analysis that the benefits were largely attributable to the prophylactic use of low-dose corticosteroids (≤20 mg/kg hydrocortisone), and these benefits were not observed at higher corticosteroids doses (Table 2). Low-dose corticosteroids prophylaxis was associated with a significantly reduced mechanical ventilation time (MD: −2.74 h, 95% CI: −4.14, −1.33 h; *p* = 0.0001, *I*^2 ^= 92%) (Figure 5), without increased risks of myocardial infarction (OR: 0.96, 95% CI: 0.43–2.17; *p* = 0.93, *I*^2 ^= 0%) (Figure 2) or insulin infusion (OR: 1.72, 95% CI: 0.83–3.55; *p* = 0.15, *I*^2 ^= 36%) (*Supplementary Figure S7*). Pooled analysis with meta-regression revealed that corticosteroids dose was significantly related to the variation in the mechanical ventilation time (exp: 1.004, 95% CI: 1.002–1.006; *p* < 0.0001), but not the variation in the other clinical outcomes (Supplementary Figures S18–S26). Funnel plots failed to reveal evidence of publication bias regarding mortality, myocardial infarction, pulmonary complications, kidney injury, postoperative infection, and neurological complications (stroke) (Supplementary Figures S27–S32). However, the funnel plots suggested that there might be some publication bias regarding new atrial fibrillation, mechanical ventilation time, and hyperglycemia requiring insulin infusion (Supplementary Figures S33–S35). Thus, we used the trim and fill method to adjust the analysis, which did not significantly alter the findings.

During pediatric cardiac surgery with CPB, corticosteroids prophylaxis was associated with a decreased CPB time (MD: −11.54 min, 95% CI: −14.32, −8.75 min; *p* < 0.001, *I*^2 ^= 5%) and an increased insulin infusion (OR: 3.68, 95% CI: 1.53–8.84; *p* = 0.004, *I*^2 ^= 48%), but did not significantly influence mortality, kidney injury, ECMO use, postoperative infection, mechanical ventilation time, and ICU length of stay [LOS] (Tables 2, 3 and Supplementary Figures S36–S43). Relative to placebo and higher dose corticosteroids (>50 mg/kg hydrocortisone), corticosteroids prophylaxis (≤50 mg/kg hydrocortisone) significantly reduced the risk of kidney injury (OR: 0.29, 95% CI: 0.09–0.96; *p* = 0.04, *I*^2 ^= 49%) (Table 2 and Supplementary Figure S39). Meta-regression revealed that corticosteroids dose was not related to the variations in mortality (exp: 0.998, 95% CI: 0.981–1.015; p = 0.734) or the duration of CPB (exp: 1.000, 95% CI: 0.993–1.008; *p* = 0.89) (Supplementary Figures S44, S45). The funnel plots failed to reveal evidence of publication bias regarding mortality, kidney injury, postoperative infection, and ICU LOS (Supplementary Figures S46–S49). However, the funnel plots suggested that there might be some publication bias regarding mechanical ventilation time and CPB duration (Supplementary Figures S50–S51). Thus, we used the trim and fill method to adjust the analysis, which did not significantly alter the findings.

## Discussion

This meta-analysis revealed that corticosteroids prophylaxis during cardiac surgery with CPB was associated with significantly decreased blood inflammatory factor concentrations of CRP, TNF-α, IL-6, and IL-8. During adult cardiac surgery, corticosteroids prophylaxis reduced the risks of postoperative atrial fibrillation and re-intubation, shortened the ICU and hospital LOSs, and reduced postoperative bleeding, although it was associated with increased risks of myocardial infarction and hyperglycemia requiring insulin infusion. Interestingly, the benefits among adult patients were largely attributable to low-dose corticosteroids use (≤20 mg/kg hydrocortisone), as the benefits were not observed among patients who received higher corticosteroids doses. In addition, low-dose corticosteroids significantly reduced the mechanical ventilation time without increasing the risks of myocardial infarction and insulin infusion, while high-dose corticosteroids were associated with increased risks of myocardial infarction and prolonged mechanical ventilation. During pediatric cardiac surgery, corticosteroids prophylaxis was associated with a shortened CPB time, an increased risk of insulin infusion, and no substantial changes in terms of mortality, ECMO use, postoperative infection, mechanical ventilation time, and ICU LOS. Moreover, corticosteroids prophylaxis (≤50 mg/kg hydrocortisone) significantly reduced the risk of kidney injury in pediatric patients.

The SIRS plays a vital role in the development of complications after cardiac surgery with CPB (1). Corticosteroids can effectively inhibit SIRS and reduce inflammatory factor concentrations, which provides a theoretical basis for prophylactic administration during cardiac surgery with CPB (2–10). However, several RCTs have indicated that corticosteroids prophylaxis did not provide significant benefits to patients undergoing cardiac surgery with CPB, and was instead associated with an increased risk of myocardial infarction and prolonged mechanical ventilation (10, 13, 14, 19). Thus, the adult cardiac surgery guidelines, as well as routine practice for adult and pediatric cardiac surgery with CPB, involve limited or no prophylactic corticosteroids (16). However, corticosteroids exert dose-dependent anti-inflammatory effects and clinical side effects (3, 4, 7). Thus, we hypothesized that an appropriate dosage range might effectively inhibit SIRS and provide clinical benefits without major side effects, as the optimal corticosteroids dose would protect cardiomyocytes rather than damage them.

Our results revealed that corticosteroids prophylaxis reduced the blood concentrations of various inflammatory markers after cardiac surgery, including CRP, TNF-α, IL-6, and IL-8. These findings support the prophylactic administration of corticosteroids to prevent SIRS after cardiac surgery with CPB (8–10). However, we did not detect any significant change in mortality, which is consistent with the results of previous studies (5–7, 13, 14, 19). This may be related to advanced cardiac surgery management and active treatment of complications in the current era.

The SIRS trial and Ho et al.’s meta-analysis of 50 small RCTs revealed that corticosteroids prophylaxis in adults significantly increased the risks of myocardial infarction and hyperglycemia requiring insulin infusion (6, 14). In this context, high doses of corticosteroids can rapidly and significantly induce insulin resistance, reduce cellular utilization of glucose, and cause hyperglycemia (20). Hyperglycemia downregulates glyoxalase 1 and glyoxalase 2, which inhibits the post-injury repair of cardiomyocytes (21). This may be the main mechanism through which high-dose corticosteroids induce myocardial infarction. We found that corticosteroids (>20 mg/kg hydrocortisone), but not low-dose corticosteroids, increased the risk of myocardial infarction and hyperglycemia requiring insulin infusion in adults. This may be because low-dose corticosteroids inhibit SIRS and protect cardiomyocytes, without substantially impairing glucose utilization. We did not observe a substantial change in this relationship when we re-analyzed data from 18 high-quality RCTs (Jadad score of ≥4, 18/25 trials), which all adopted the general definition of myocardial infarction and used cardiac biomarkers to predict its occurrence. In children, corticosteroids increased the use of insulin but did not significantly influence the risk of myocardial infarction, which may be related to neonatal cardiomyocytes having increased glucose uptake and utilization (22).

The DECS trial (13) and the SIRS trial (14) revealed that corticosteroids prophylaxis did not reduce the risk of atrial fibrillation in adult patients after cardiac surgery. However, meta-analyses by Ho et al. (6) and Ng et al. (7) revealed that corticosteroids prophylaxis could significantly reduce the incidence of atrial fibrillation. Ho et al. (6) reported that both low-dose and high-dose corticosteroids could significantly reduce the risk of atrial fibrillation. Ng et al.’s meta-analysis included the DECS and SIRS trials, but did not include a stratified dose analysis (7). Interestingly, we found that only low-dose corticosteroids (≤20 mg/kg hydrocortisone) were effective for reducing the risk of atrial fibrillation, with no positive effects observed at a slightly higher dose (20–40 mg/kg hydrocortisone), a high dose (40–100 mg/kg hydrocortisone), or an ultra-high dose (>100 mg/kg hydrocortisone). These findings were not noticeably different when we re-analyzed 27 high-quality RCTs (27/33 RCTs), with low heterogeneity and no detectable publication bias. Unfortunately, the relevant molecular mechanisms are not clear, although the SIRS can induce atrial fibrillation (23). Thus, we speculate that low-dose corticosteroids might inhibition SIRS without increasing cardiomyocyte damage, which would reduce the incidence of atrial fibrillation. In contrast, high-dose corticosteroids might reduce SIRS but increase cardiomyocyte damage, which would not reduce the risk of atrial fibrillation.

The SIRS can cause systemic multi-organ damage, which often involves kidney damage (5–7). Prophylactic administration of corticosteroids protects the tissues and organs by inhibiting SIRS and thus reduces complications (8–10). We failed to identify significant effects of corticosteroids prophylaxis on the risks of pulmonary complications, neurological complications (stroke), gastrointestinal bleeding, and delirium, which might be related to the low incidences of those outcomes. However, prophylactic corticosteroids (≤50 mg/kg hydrocortisone) significantly reduced the risk of kidney injury in pediatric patients, and low-dose corticosteroids (≤20 mg/kg hydrocortisone) might reduce the risk of kidney injury in adult patients. While corticosteroids suppress the normal immune response and may increase the risk of postoperative infection (7), our results and those from previous studies suggest that corticosteroids prophylaxis did not influence the risk of postoperative infection (6, 13, 14). Ho et al. (6) reported that corticosteroids prophylaxis was closely associated with prolonged mechanical ventilation, and we found that low-dose corticosteroids (≤20 mg/kg hydrocortisone) significantly shortened the mechanical ventilation duration for adult patients, while high doses (>100 mg/kg hydrocortisone) significantly prolonged the mechanical ventilation duration. We also found that low-dose corticosteroids significantly reduced the ICU and hospital LOSs for adult patients, which might be related to accelerated recovery that was caused by suppression of the SIRS and reduced tissue and organ damage. Therefore, corticosteroids may be a cost-effective prophylactic treatment (generally <$5/patient) that can help reduce the burden on patients and hospitals by decreasing the risks of complications and shortening the ICU and hospital LOSs. Furthermore, the lower risk of complications may improve patients’ perioperative quality of life.

Our meta-analysis considered the dose-dependent benefits and risks of prophylactic corticosteroids during adult and pediatric cardiac surgery based on 29 clinical outcomes. Our findings conflict with the lack of support for corticosteroids prophylaxis during cardiac surgery in previous studies (5–7, 13, 14) and the guidelines for adult cardiac surgery (16). Our results suggest that low-dose corticosteroids (≤20 mg/kg hydrocortisone) were not associated with a significant reduction in mortality, but might substantially benefit adult patients by inhibiting SIRS and reducing complications. Therefore, we recommend prophylactic administration of low-dose corticosteroids (≤20 mg/kg hydrocortisone) during adult cardiac surgery. However, the optimal dose range for corticosteroids prophylaxis during pediatric cardiac surgery is unclear, as we only identified a small number of related RCTs. Nevertheless, our results indicate that high-dose glucocorticoids did not provide any benefits and significantly increased insulin use, which may increase the risk of hyperglycemia and related complications.

The evidence from our study was judged to be high based on the GRADE system. The low-dose subgroup for adult cardiac surgery (≤20 mg/kg hydrocortisone) only included 14 small RCTs, although 10 of these RCTs were considered high-quality based on the Jadad scores. Thus, large multi-center RCTs are needed as an additional source of evidence to clarify efficacy and optimal dose range for low-dose prophylactic corticosteroids during adult and pediatric cardiac surgery with CPB.

## Data Availability

The original contributions presented in the study are included in the article/Supplementary Material, further inquiries can be directed to the corresponding author/s.
