# Supplementary material for: Meta-Analysis: Shouldn’t Prophylactic Corticosteroids be Administered During Cardiac Surgery with Cardiopulmonary Bypass?
Source: Front Surg. 2022 Jun 1;9:832205. doi: 10.3389/fsurg.2022.832205 (PMC9198450; doi:10.3389/fsurg.2022.832205)
Supplement: Supplementary file 1 [file Table_6_v1.docx]

**eTable 1. search terms**

**eFigure 1. Impact of corticosteroids on hyperglycemia requiring insulin infusion (adult)**

**eFigure 2. Impact of corticosteroids on LOS in ICU (days) (adult)**

**eFigure 3. Impact of corticosteroids on LOS in hospital (days) (adult)**

**eFigure 4. Impact of corticosteroids on postoperative bleeding (mL)(adult)**

**eFigure 5. Impact of corticosteroids on re-intubation (adult)**

**eFigure 6. Impact of corticosteroids on IL-6 concentrations at 24 hours (pg/ml) (adult)**

**eFigure 7. Impact of corticosteroids on TNF-α concentrations at 24 hours (pg/ml) (adult)**

**eFigure 8. Impact of corticosteroids on IL-8 concentrations at 24 hours (pg/ml) (adult)**

**eFigure 9. Impact of corticosteroids on highest/24h CRP concentrations (μg/ml)(pediatric)**

**eFigure 10. Impact of corticosteroids on highest/24h IL-6 concentrations (pg/ml) (pediatric)**

**eFigure 11. Impact of corticosteroids on highest/24h IL-10 concentrations (pg/ml) (pediatric)**

**eFigure 12. Impact of corticosteroids on kidney injury (adult)**

**eFigure 13. Impact of corticosteroids on pulmonary complications (adult)**

**eFigure 14. Impact of corticosteroids on neurological complications (strok)(adult)**

**eFigure 15. Impact of corticosteroids on gastro-intestinal bleeding (adult)**

**eFigure 16. Impact of corticosteroids on postoperative infection (adult)**

**eFigure 17. Impact of corticosteroids on delirium (adult)**

**eFigure 18. Meta-regression of doses of corticosteroids and reduction**

**in mortality risk for adult cardiac surgery with CPB**

**eFigure 19. Meta-regression of doses of corticosteroids and reduction**

**in new atrial fibrillation risk for adult cardiac surgery with CPB**

**eFigure 20. Meta-regression of doses of corticosteroids and reduction**

**in myocardial infarction risk for adult cardiac surgery with CPB**

**eFigure 21. Meta-regression of doses of corticosteroids and reduction**

**in pulmonary complications risk for adult cardiac surgery with CPB**

**eFigure 22. Meta-regression of doses of corticosteroids and reduction in kidney injury risk for adult cardiac surgery with CPB**

**eFigure 23. Meta-regression of doses of corticosteroids and reduction**

**in postoperative infection risk for adult cardiac surgery with CPB**

**eFigure 24. Meta-regression of doses of corticosteroids and reduction in neurological complications (strok) risk for adult cardiac surgery with CPB**

**eFigure 25. Meta-regression of doses of corticosteroids and reduction**

**in hyperglycemia requiring insulin infusion risk for adult cardiac surgery with CPB**

**eFigure 26. Meta-regression of doses of corticosteroids and reduction in LOS in ICU for adult cardiac surgery with CPB**

**eFigure 27. Funnel plot of mortality for adult cardiac surgery with CPB**

**eFigure 28. Funnel plot of myocardial infarction for adult cardiac surgery with CPB**

**eFigure 29. Funnel plot of pulmonary complications for adult cardiac surgery with CPB**

**eFigure 30. Funnel plot of kidney injury for adult cardiac surgery with CPB**

**eFigure 31. Funnel plot of postoperative infection for adult cardiac surgery with CPB**

**eFigure 32. Funnel plot of neurological complications (strok) for adult cardiac surgery with CPB**

**eFigure 33. Funnel plot of new atrial fibrillation for adult cardiac surgery with CPB**

**eFigure 34. Funnel plot of mechanical ventilation time for adult cardiac surgery with CPB**

**eFigure 35. Funnel plot of hyperglycemia requiring insulin infusion for adult cardiac surgery with CPB**

**eFigure 36. Impact of corticosteroids on duration of CPB (minutes) (pediatric)**

**eFigure 37. Impact of corticosteroids on hyperglycemia requiring insulin infusion (pediatric)**

**eFigure 38. Impact of corticosteroids on mortality (pediatric)**

**eFigure 39. Impact of corticosteroids on kidney injury (pediatric)**

**eFigure 40. Impact of corticosteroids on postoperative ECMO use (pediatric)**

**eFigure 41. Impact of corticosteroids on postoperative infection (pediatric)**

**eFigure 42. Impact of corticosteroids on mechanical ventilation time (pediatric)**

**eFigure 43. Impact of corticosteroids on LOS in ICU (pediatric)**

**eFigure 44. Meta-regression of doses of corticosteroids and**

**reduction in mortality risk for pediatric cardiac surgery with CPB**

**eFigure 45. Meta-regression of doses of corticosteroids and reduction**

**in duration of CPB for pediatric cardiac surgery with CPB**

**eFigure 46. Funnel plot of mortality for pediatric cardiac surgery with CPB**

**eFigure 47. Funnel plot of kidney injury for pediatric cardiac surgery with CPB**

**eFigure 48. Funnel plot of postoperative infection for pediatric cardiac surgery with CPB**

**eFigure 49. Funnel plot of LOS in ICU for pediatric cardiac surgery with CPB**

**eFigure 50. Funnel plot of mechanical ventilation time for pediatric cardiac surgery with CPB**

**eFigure 51. Funnel plot of duration of CPB for pediatric cardiac surgery with CPB**

**eTable 1. search terms**

| Pubmed | Cochrane Central Register of Controlled Trials | Embase |
| --- | --- | --- |
| (((Corticosteroids[MeSH Terms]) OR ((Hormones, Adrenal Cortex[Title/Abstract]) OR (Corticosteroids[Title/Abstract])) OR (Corticoid[Title/Abstract])) OR (Corticoids[Title/Abstract])) OR (Metacortandracin[Title/Abstract])) OR (Glucocorticoids[Title/Abstract])) OR (17-Ketosteroids[Title/Abstract])) OR (Catatoxic Steroids[Title/Abstract])) OR (Steroids, Catatoxic[Title/Abstract])) OR (Steroids[Title/Abstract])) OR (Dehydrocortisone[Title/Abstract])) OR (Delta-Cortisone[Title/Abstract])) OR (Metacortandracin[Title/Abstract])) OR (Prednison[Title/Abstract])) OR (Meprednisone[Title/Abstract])) OR (Alpha-methylprednisolone[Title/Abstract])) OR (Methylprednisolone[Title/Abstract])) OR (Metipred[Title/Abstract])) OR (6-Methylprednisolone[Title/Abstract])) OR (6 Methylprednisolone[Title/Abstract])) OR (Urbason[Title/Abstract])) OR (Medrol[Title/Abstract])) OR (Cortisol[Title/Abstract])) OR (Hydrocortisone[Title/Abstract])) OR (Hydrocortiso[Title/Abstract])) OR (Epicortisol[Title/Abstract])) OR (Cortifair[Title/Abstract])) OR (Cortril[Title/Abstract])) OR (Methylfluorprednisolone[Title/Abstract])) OR (Hexadecadrol[Title/Abstract])) OR (Decameth[Title/Abstract])) OR (Decaspray[Title/Abstract])) OR (Dexasone[Title/Abstract])) OR (Dexpak[Title/Abstract])) OR (Maxidex[Title/Abstract])) OR (Millicorten[Title/Abstract])) OR (Oradexon[Title/Abstract])) OR (Hexadrol[Title/Abstract])) OR (Anti-inflammator[Title/Abstract])) OR (Anti next inflammator[Title/Abstract])) OR (Antiinflammator[Title/Abstract])) OR (Antiflogistic[Title/Abstract]))) AND ((Extracorporeal Circulations[MeSH Terms]) OR ((((((((((((((((((((((((Circulation, Extracorporeal[Title/Abstract]) OR (Circulations, Extracorporeal[Title/Abstract])) OR (Extracorporeal Circulations[Title/Abstract])) OR (Heart-Lung Bypass[Title/Abstract])) OR (Bypass, Heart-Lung[Title/Abstract])) OR (Bypasses, Heart-Lung[Title/Abstract])) OR (Heart Lung Bypass[Title/Abstract])) OR (Heart-Lung Bypasses[Title/Abstract])) OR (Bypass, Cardiopulmonary[Title/Abstract])) OR (Bypasses, Cardiopulmonary[Title/Abstract])) OR (Cardiopulmonary Bypasses[Title/Abstract])) OR (Oxygenation, Extracorporeal Membrane[Title/Abstract])) OR (Extracorporeal Membrane Oxygenations[Title/Abstract])) OR (Membrane Oxygenation, Extracorporeal[Title/Abstract])) OR (Membrane Oxygenations, Extracorporeal[Title/Abstract])) OR (Oxygenations, Extracorporeal Membrane[Title/Abstract])) OR (Bypass, Left Heart[Title/Abstract])) OR (Bypasses, Left Heart[Title/Abstract])) OR (Heart Bypasses, Left[Title/Abstract])) OR (Left Heart Bypasses[Title/Abstract])) OR (Heart Bypasses[Title/Abstract])) OR (Left Heart Bypass[Title/Abstract])) OR (CPB[Title/Abstract])) OR (Coronary Artery Bypass[Title/Abstract])))) AND (randomized controlled trial[Publication Type] OR randomized[Title/Abstract] OR placebo[Title/Abstract]) | ID Search Hits  #1 MeSH descriptor: [Adrenal Cortex Hormones] explode all trees 14177  #2 (Hormones, Adrenal Cortex):ti,ab,kw OR (Corticosteroids):ti,ab,kw OR (Corticoid):ti,ab,kw OR (Corticoids):ti,ab,kw OR (Metacortandracin):ti,ab,kw (Word variations have been searched) 22086  #3 (Glucocorticoids):ti,ab,kw OR (17Ketosteroids):ti,ab,kw OR (Catatoxic Steroids):ti,ab,kw OR (Steroids, Catatoxic):ti,ab,kw OR (Dehydrocortisone):ti,ab,kw (Word variations have been searched) 8315  #4 ("delta-cortisone"):ti,ab,kw OR (metacortandracin):ti,ab,kw OR (Prednison):ti,ab,kw OR (Meprednisone):ti,ab,kw OR (alpha-methylprednisolone):ti,ab,kw (Word variations have been searched) 9363  #5 (Methylprednisolone):ti,ab,kw OR (Metipred):ti,ab,kw OR (Medrol):ti,ab,kw OR (6 Methylprednisolone):ti,ab,kw OR (Urbason):ti,ab,kw (Word variations have been searched) 5218  #6 (Cortisol):ti,ab,kw OR (Hydrocortisone):ti,ab,kw OR (Hydrocortiso):ti,ab,kw OR (Epicortisol):ti,ab,kw OR (Cortifair):ti,ab,kw (Word variations have been searched) 13583  #7 (Cortril):ti,ab,kw OR (Methylfluorprednisolone):ti,ab,kw OR (Hexadecadrol):ti,ab,kw OR (Decameth):ti,ab,kw OR (Decaspray):ti,ab,kw (Word variations have been searched) 7  #8 (Dexasone):ti,ab,kw OR (Dexpak):ti,ab,kw OR (Maxidex):ti,ab,kw OR (Millicorten):ti,ab,kw OR (Oradexon):ti,ab,kw (Word variations have been searched) 40  #9 (Hexadrol):ti,ab,kw (Word variations have been searched) 3  #10 #1 OR #2 OR #3 OR #4 OR #5 OR #6 OR #7 OR #8 OR #9 50918  #11 MeSH descriptor: [Anti-Inflammatory Agents] explode all trees 13130  #12 (Anti-inflammator):ti,ab,kw OR (Anti next inflammator):ti,ab,kw OR (Antiinflammator):ti,ab,kw OR (Antiflogistic):ti,ab,kw OR (Antiphlogistic):ti,ab,kw (Word variations have been searched) 431  #13 #11 OR #12 13512  #14 #10 OR #13 61249  #15 MeSH descriptor: [Extracorporeal Circulation] explode all trees 3992  #16 (Circulation, Extracorporeal):ti,ab,kw OR (Circulations, Extracorporeal):ti,ab,kw OR (Extracorporeal Circulations):ti,ab,kw OR (Heart-Lung Bypass):ti,ab,kw OR (Bypass, Heart-Lung):ti,ab,kw (Word variations have been searched) 1475  #17 (Bypasses, Heart-Lung):ti,ab,kw OR (Heart Lung Bypass):ti,ab,kw OR (Heart-Lung Bypasses):ti,ab,kw OR (Bypass, Cardiopulmonary):ti,ab,kw OR (Bypasses, Cardiopulmonary):ti,ab,kw (Word variations have been searched) 6971  #18 (Cardiopulmonary Bypasses):ti,ab,kw OR (Oxygenation, Extracorporeal Membrane):ti,ab,kw OR (Extracorporeal Membrane Oxygenations):ti,ab,kw OR (Membrane Oxygenation, Extracorporeal):ti,ab,kw OR (Membrane Oxygenations, Extracorporeal):ti,ab,kw (Word variations have been searched) 7086  #19 (Oxygenations, Extracorporeal Membrane):ti,ab,kw OR (CPB):ti,ab,kw (Word variations have been searched) 3585  #20 #15 OR #16 OR #17 OR #18 OR #19 9350  #21 MeSH descriptor: [Cardiac Surgical Procedures] explode all trees 12642  #22 (Coronary Artery Bypass):ti,ab,kw (Word variations have been searched) 12294  #23 (Bypass, Left Heart):ti,ab,kw OR (Bypasses, Left Heart):ti,ab,kw OR (Heart Bypasses, Left):ti,ab,kw OR (Left Heart Bypasses):ti,ab,kw OR (Left Heart Bypass):ti,ab,kw (Word variations have been searched) 2008  #24 #21 OR #22 OR #23 19306  #25 #20 OR #24 23822  #26 #14 AND #25 767 | No. Query Results Results  #76. #45 AND #71 AND #75 707  #75. #72 OR #73 OR #74 680,373  #74. 'double-blind':ab,ti 193,969  #73. 'placebo':ab 304,986  #72. 'random':ab,ti 322,113  #71. #46 OR #47 OR #48 OR #49 OR #50 OR #51 OR #52 OR #53 OR #54 OR #55 OR #56 OR #57 OR #58 OR #59 OR #60 OR #61 OR #62 OR #63 OR #64 OR #65 OR #66 OR #67 OR #68 OR #69 OR #70 153,961  #70. 'cpb':ab,ti 15,041  #69. 'coronary artery bypass':ab,ti 53,271  #68. 'left heart bypass':ab,ti 567  #67. 'left heart bypasses':ab,ti 5  #66. 'heart bypasses, left':ab,ti  #65. 'bypasses, left heart':ab,ti  #64. 'bypass, left heart':ab,ti 4  #63. 'oxygenations, extracorporeal membrane':ab,ti  #62. 'membrane oxygenations, extracorporeal':ab,ti  #61. 'membrane oxygenation, extracorporeal':ab,ti 23  #60. 'extracorporeal membrane oxygenations':ab,ti 8  #59. 'oxygenation, extracorporeal membrane':ab,ti 13  #58. 'cardiopulmonary bypasses':ab,ti 18  #57. 'bypasses, cardiopulmonary':ab,ti  #56. 'bypass, cardiopulmonary':ab,ti 27  #55. 'heart-lung bypasses':ab,ti  #54. 'heart lung bypass':ab,ti 132  #53. 'bypasses, heart-lung':ab,ti  #52. 'bypass, heart-lung':ab,ti 3  #51. 'heart-lung bypass':ab,ti 132  #50. 'extracorporeal circulations':ab,ti 30  #49. 'circulations, extracorporeal':ab,ti  #48. 'circulation, extracorporeal':ab,ti 14  #47. 'coronary artery bypass graft'/exp 73,495  #46. 'extracorporeal circulation'/exp 71,015  #45. #1 OR #2 OR #3 OR #4 OR #5 OR #6 OR #7 OR #8 OR 2,783,513  #9 OR #10 OR #11 OR #12 OR #13 OR #14 OR #15 OR #16 OR #17 OR #18 OR #19 OR #20 OR #21 OR #22 OR #23 OR #24 OR #25 OR #26 OR #27 OR #28 OR #29 OR #30 OR #31 OR #32 OR #33 OR #34 OR #35 OR #36 OR #37 OR #38 OR #39 OR #40 OR #41 OR #42 OR #43 OR #44 #44. 'antiphlogistic':ab,ti 1,334  #43. 'antiflogistic':ab,ti 37  #42. 'antiinflammator':ab,ti 3  #41. 'anti next inflammator':ab,ti  #40. 'anti-inflammator':ab,ti 8  #39. 'hexadrol':ab,ti 7  #38. 'oradexon':ab,ti 11  #37. 'millicorten':ab,ti 5  #36. 'maxidex':ab,ti 36  #35. 'dexpak':ab,ti  #34. 'dexasone':ab,ti 12  #33. 'decaspray':ab,ti 1  #32. 'decameth':ab,ti  #31. 'hexadecadrol':ab,ti 40  #30. 'methylfluorprednisolone':ab,ti 1  #29. 'cortril':ab,ti 17  #28. 'cortifair':ab,ti  #27. 'epicortisol':ab,ti 26  #26. 'hydrocortiso':ab,ti 1  #25. 'hydrocortisone':ab,ti 21,810  #24. 'cortisol':ab,ti 77,701  #23. 'medrol':ab,ti 608  #22. 'urbason':ab,ti 58  #21. '6 methylprednisolone':ab,ti 211  #20. '6-methylprednisolone':ab,ti 211  #19. 'metipred':ab,ti 20  #18. 'methylprednisolone':ab,ti 25,223  #17. 'alpha-methylprednisolone':ab,ti 40  #16. 'meprednisone':ab,ti 53  #15. 'prednison':ab,ti 271  #14. 'metacortandracin':ab,ti 72  #13. 'delta-cortisone':ab,ti 91  #12. 'dehydrocortisone':ab,ti 39  #11. 'steroids, catatoxic':ab,ti  #10. 'catatoxic steroids':ab,ti 54  #9. '17-ketosteroids':ab,ti 2,201  #8. 'glucocorticoids':ab,ti 43,953  #7. 'metacortandracin':ab,ti 72  #6. 'corticoids':ab,ti 4,596  #5. 'corticoid':ab,ti 4,110  #4. 'corticosteroids':ab,ti 102,571  #3. 'hormones, adrenal cortex':ab,ti 1  #2. 'antiinflammatory agent'/exp 1,977,532  #1. 'steroid'/exp 1,600,448 |

**References**

1 Abbaszadeh M, Khan ZH, Mehrani F, Jahanmehr H. Perioperative intravenous corticosteroids reduce incidence of atrial fibrillation following cardiac surgery: A randomized study. *Brazilian Journal of Cardiovascular Surgery* 2012; 27(1): 18-23.

2 Abd El-Hakeem EE, Ashry MA, El-Minshawy A, Maghraby EA. Influence of dexamethasone on cytokine balance in patients undergoing valve replacement surgery. *Egyptian Journal of Anaesthesia* 2003; 19(3): 205-14.

3 Al-Shawabkeh Z, Al-Nawaesah K, Anzeh RA, Al-Odwan H, Al-Rawashdeh WAB, Altaani H. Use of short-term steroids in the prophylaxis of atrial fibrillation after cardiac surgery. *Journal of the Saudi Heart Association* 2017; 29(1): 23‐9.

4 Amanullah MM, Hamid M, Hanif HM, et al. Effect of steroids on inflammatory markers and clinical parameters in congenital open heart surgery: a randomised controlled trial. *Cardiology in the young* 2016; 26(3): 506-15.

5 LW A, L B, BS T, JP R. Effect of methylprednisolone on endotoxemia and complement activation during cardiac surgery. *Journal of cardiothoracic anesthesia* 1989; 3(5): 544-9.

6 M A, IS P, N W, Y T. Steroid supplementation: a legitimate pharmacotherapy after neonatal open heart surgery. *The Annals of thoracic surgery* 2005; 80(5): 1672-8.

7 H B, F C, A B, et al. The effect of oral prednisolone with chronic obstructive pulmonary disease undergoing coronary artery bypass surgery. *Journal of cardiac surgery* 2005; 20(3): 252-6.

8 MJ B, VM Y, MA T, JS C. Complement activation during cardiopulmonary bypass: quantitative study of effects of methylprednisolone and pulsatile flow. *British medical journal (Clinical research ed)* 1983; 287(6407): 1747-50.

9 A B, M V, P L, et al. The effect of methylprednisolone treatment on the cardiopulmonary bypass-induced systemic inflammatory response. European journal of cardio-thoracic surgery : official journal of the *European Association for Cardio-thoracic Surgery* 2004; 26(5): 932-8.

10 Brettner F, Chappell D, Nebelsiek T, et al. Preinterventional hydrocortisone sustains the endothelial glycocalyx in cardiac surgery. *Clinical hemorheology and microcirculation* 2019; 71(1): 59-70.

11 Bronicki RA, Backer CL, Baden HP, Mavroudis C, Crawford SE, Green TP. Dexamethasone reduces the inflammatory response to cardiopulmonary bypass in children. *Annals of Thoracic Surgery* 2000; 69(5): 1490-5.

12 Butler J, Pathi VL, Paton RD, et al. Acute-phase responses to cardiopulmonary bypass in children weighing less than 10 kilograms. *The Annals of thoracic surgery* 1996; 62(2): 538-42.

13 NC C, JR P, HV S, et al. Complement activation during cardiopulmonary bypass. Comparison of bubble and membrane oxygenators. *The Journal of thoracic and cardiovascular surgery* 1986; 91(2): 252-8.

14 Celik JB, Gormus N, Okesli S, Gormus ZI, Solak H. Methylprednisolone prevents inflammatory reaction occurring during cardiopulmonary bypass: effects on TNF-alpha, IL-6, IL-8, IL-10. *Perfusion* 2004; 19(3): 185-91.

15 MA C, MP N, B B, M B, S S. Pulmonary effects of methylprednisolone in patients undergoing coronary artery bypass grafting and early tracheal extubation. *Anesthesia and analgesia* 1998; 87(1): 27-33.

16 MA C, MP N, BP B, M B, S S. Hemodynamic effects of methylprednisolone in patients undergoing cardiac operation and early extubation. *The Annals of thoracic surgery* 1999; 67(4): 1006-11.

17 MA C, RA D-A, MP N, BP B, M B. Methylprednisolone does not benefit patients undergoing coronary artery bypass grafting and early tracheal extubation. *The Journal of thoracic and cardiovascular surgery* 2001; 121(3): 561-9.

18 Checchia PA, Backer CL, Bronicki RA, et al. Dexamethasone reduces postoperative troponin levels in children undergoing cardiopulmonary bypass*. *Critical care medicine* 2003; 31(6): 1742-5.

19 Codd JE, Wiens RD, Barner HB. Steroids and myocardial preservation. *Journal of thoracic and cardiovascular surgery* 1977; 74(3): 418‐22.

20 Coetzer M, Coetzee A, Rossouw G. The effect of methylprednisolone, given prior to cardiopulmonary bypass, on indices of gas exchange. *Cardiovascular journal of southern africa* 1996; 86(4): C188‐C92.

21 Danielson M, Reinsfelt B, Westerlind A, Zetterberg H, Blennow K, Ricksten SE. Effects of methylprednisolone on blood-brain barrier and cerebral inflammation in cardiac surgery-a randomized trial. *Journal of neuroinflammation* 2018; 15(1): 283.

22 Demir T, Demir H, Tansel T, et al. Influence of Methylprednisolone on Levels of Neuron-Specific Enolase in Cardiac Surgery: A Corticosteroid Derivative to Decrease Possible Neuronal Damage. *Journal of cardiac surgery* 2009; 24(4): 397-403.

23 Demir T, Ergenoglu MU, Demir HB, et al. Pretreatment with methylprednisolone improves myocardial protection during on-pump coronary artery bypass surgery. *The heart surgery forum* 2015; 18(4): E171-7.

24 Dieleman JM, Nierich AP, Rosseel PM, et al. Intraoperative high-dose dexamethasone for cardiac surgery: a randomized controlled trial. *Jama* 2012; 308(17): 1761‐7.

25 El ASR, Rosseel PMJ, De LJJ, et al. Dexamethasone decreases the pro‐ to anti‐inflammatory cytokine ratio during cardiac surgery. *British journal of anaesthesia* 2002; (4): 4.

26 Enc Y, Karaca P, Ayoglu U, Camur G, Kurc E, Cicek S. The acute cardioprotective effect of glucocorticoid in myocardial ischemia-reperfusion injury occurring during cardiopulmonary bypass. *Heart and vessels* 2006; 21(3): 152-6.

27 RM E, JA R, JE F, DW D, R K, DK D. Influence of steroids on complement and cytokine generation after cardiopulmonary bypass. *The Annals of thoracic surgery* 1995; 60(3): 801-4.

28 Fecht DC, Magovern GJ, Park SB, et al. Beneficial effects of methylprednisolone in patients on cardiopulmonary bypass. *Circulatory shock* 1978; 5(4): 415-22.

29 Ferries LH, Jr JJM, Iii JFR. The effect of methylprednisolone on complement activation during cardiopulmonary bypass. *Journal of Extra Corporeal Technology* 1984; 16(3): 83-8.

30 Fillinger MP, Rassias AJ, Guyre PM, et al. Glucocorticoid effects on the inflammatory and clinical responses to cardiac surgery. *Journal of cardiothoracic and vascular anesthesia* 2002; 16(2): 163-9.

31 Giomarelli, Scolletta, Borrelli, Biagioli. Myocardial and Lung Injury After Cardiopulmonary Bypass: Role of Interleukin (IL)-10. *Annals of Thoracic Surgery* 2003; 76: 117-23.

32 Gomez Polo JC, Vilacosta I, Gomez-Alvarez Z, et al. Short term use of corticosteroids in the prophylaxis of atrial fibrillation after cardiac surgery and impact on the levels of acute phase proteins in this context. *European heart journal* 2018; 39: 668-9.

33 Graham E, Martin R, Buckley J, et al. Corticosteroid Therapy in Neonates Undergoing Cardiopulmonary Bypass: Randomized Controlled Trial. *Journal of the American College of Cardiology* 2019; 74(5): 659-68.

34 Halonen J, Halonen P, Järvinen O, et al. Corticosteroids for the prevention of atrial fibrillation after cardiac surgery: a randomized controlled trial. *Jama* 2007; 297(14): 1562-7.

35 Halvorsen P, Raeder J, White PF, et al. The effect of dexamethasone on side effects after coronary revascularization procedures. *Anesthesia and analgesia* 2003; 96(6): 1578‐83.

36 Hao X, Han J, Zeng H, et al. The effect of methylprednisolone prophylaxis on inflammatory monocyte subsets and suppressive regulatory T cells of patients undergoing cardiopulmonary bypass. *Perfusion* 2019; 34(5): 364-74.

37 Harig F, Feyrer R, Mahmoud F, Blum U, Von dE, J. Reducing the Post-Pump Syndrome by Using Heparin-Coated Circuits, Steroids, or Aprotinin. *The Thoracic and cardiovascular surgeon* 1999; 47(02): 111-8.

38 Harig F, Hohenstein B, Jürgen VDE, Weyand M. MODULATING IL-6 AND IL-10 LEVELS BY PHARMACOLOGIC STRATEGIES AND THE IMPACT OF DIFFERENT EXTRACORPOREAL CIRCULATION PARAMETERS DURING CARDIAC SURGERY. *Shock* 2001; 16: 33-8.

39 R H, E W, K S, et al. Dexamethasone pretreatment provides antiinflammatory and myocardial protection in neonatal arterial switch operation. *The Annals of thoracic surgery* 2012; 93(3): 869-76.

40 NJ J, W vO, L vdB, et al. Inhibition by dexamethasone of the reperfusion phenomena in cardiopulmonary bypass. *The Journal of thoracic and cardiovascular surgery* 1991; 102(4): 515-25.

41 J K-N, E P, KT O, et al. Methylprednisolone in neonatal cardiac surgery: reduced inflammation without improved clinical outcome. *The Annals of thoracic surgery* 2013; 95(6): 2126-32.

42 Keski-Nisula J, Suominen PK, Olkkola KT, et al. Effect of Timing and Route of Methylprednisolone Administration During Pediatric Cardiac Surgical Procedures. *Annals of Thoracic Surgery* 2015; 99(1): 180-5.

43 Keski-Nisula J, Arvola O, Jahnukainen T, Andersson S, Pesonen E. Reduction of Inflammation by High-Dose Methylprednisolone Does not Attenuate Oxidative Stress in Children Undergoing Bidirectional Glenn Procedure With or Without Aortic Arch or Pulmonary Arterial Repair. *Journal of cardiothoracic and vascular anesthesia* 2020; 34(6): 1542-7.

44 Kilger E, Weis F, Briegel J, et al. Stress doses of hydrocortisone reduce severe systemic inflammatory response syndrome and improve early outcome in a risk group of patients after cardiac surgery. *Critical care medicine* 2003; 31(4): 1068-74.

45 Schelling G, Kilger E, Roozendaal B, et al. Stress doses of hydrocortisone, traumatic memories, and symptoms of posttraumatic stress disorder in patients after cardiac surgery: a randomized study. *Biol Psychiatry* 2004; 55(6): 627-33.

46 Kiliçkan L, Yumuk Z, Bayindir O. The effect of combined preinduction thoracic epidural anaesthesia and glucocorticoid administration in perioperative interleukin-10 levels and hyperglycemia. A randomized controlled trial. *Journal of Cardiovascular Surgery* 2008; 49(1): 87-93.

47 Liakopoulos OJ, Schmitto JD, Kazmaier S, et al. Cardiopulmonary and systemic effects of methylprednisolone in patients undergoing cardiac surgery. *The Annals of thoracic surgery* 2007; 84(1): 110-8.

48 L L, C F, P J, AK O. Effects of dexamethasone on clinical course, C-reactive protein, S100B protein and von Willebrand factor antigen after paediatric cardiac surgery. *British journal of anaesthesia* 2003; 90(6): 728-32.

49 BG L, RH H, AH E, et al. Effect of dexamethasone on perioperative renal function impairment during cardiac surgery with cardiopulmonary bypass. *British journal of anaesthesia* 2004; 93(6): 793-8.

50 Lomivorotov VV, Efremov SM, Kalinichenko AP, et al. Methylprednisolone Use is Associated with Endothelial Cell Activation Following Cardiac Surgery. *Heart Lung & Circulation* 2013; 22(1): 25-30.

51 Lomivorotov V, Kornilov I, Boboshko V, et al. Effect of Intraoperative Dexamethasone on Major Complications and Mortality Among Infants Undergoing Cardiac Surgery: The DECISION Randomized Clinical Trial. *Jama* 2020; 323(24): 2485-92.

52 Mardani D, Bigdelian H. The effect of dexamethasone prophylaxis on postoperative delirium after cardiac surgery: A randomized trial. *Journal of Research in Medical Sciences* 2012; 17(1 SPL.1): S113-S9.

53 Mayumi H, Zhang QW, Nakashima A, et al. Synergistic immunosuppression caused by high-dose methylprednisolone and cardiopulmonary bypass. *The Annals of thoracic surgery* 1997; 63(1): 129-37.

54 Mcbride WT, Allen S, Gormley SMC, et al. Methylprednisolone favourably alters plasma and urinary cytokine homeostasis and subclinical renal injury at cardiac surgery. *Cytokine* 2004; 27(2-3): 0-89.

55 AM M, BG L, LP A, et al. Dexamethasone: benefit and prejudice for patients undergoing on-pump coronary artery bypass grafting: a study on myocardial, pulmonary, renal, intestinal, and hepatic injury. *Chest* 2005; 128(4): 2677-87.

56 Morton JR, Hiebert CA, Lutes CA, White RL. Effect of methylprednisolone on myocardial preservation during coronary artery surgery. *American journal of surgery* 1976; 131(4): 419-22.

57 Mott AR, Fraser CD, Jr., Kusnoor AV, et al. The effect of short-term prophylactic methylprednisolone on the incidence and severity of postpericardiotomy syndrome in children undergoing cardiac surgery with cardiopulmonary bypass. *Journal of the American College of Cardiology* 2001; 37(6): 1700-6.

58 GS M, SS S, JW S, et al. Small-dose dexamethasone improves quality of recovery scores after elective cardiac surgery: a randomized, double-blind, placebo-controlled study. *Journal of cardiothoracic and vascular anesthesia* 2011; 25(6): 950-60.

59 Niazi Z, Flodin P, Joyce L, Smith J, Lillehei RC. Effects of glucocorticosteroids in patients undergoing coronary artery bypass surgery. *Chest* 1979; 76(3): 262-8.

60 Oliver WC, Jr., Nuttall GA, Orszulak TA, et al. Hemofiltration but not steroids results in earlier tracheal extubation following cardiopulmonary bypass: a prospective, randomized double-blind trial. *Anesthesiology* 2004; 101(2): 327-39.

61 K P, JG A, WR J, et al. The effects of steroids on the occurrence of postoperative atrial fibrillation after coronary artery bypass grafting surgery: a prospective randomized trial. *The Journal of thoracic and cardiovascular surgery* 2005; 130(1): 93-8.

62 Rao G, King J, Ford W, King G, Rao G. The Effects of Methylprednisolone on the Complications of Coronary Artery Surgery. *Vascular surgery* 1977; 11(1): 1-7.

63 Rubens FD, Nathan H, Labow R, et al. Effects of Methylprednisolone and a Biocompatible Copolymer Circuit on Blood Activation During Cardiopulmonary Bypass. *Annals of Thoracic Surgery* 2005; 79(2): 0-665.

64 Rumalla, Vishnu. The Effects of Glucocorticoid Therapy on Inflammatory Responses to Coronary Artery Bypass Graft Surgery. *Archives of Surgery* 2001; 136(9): 1039.

65 T S, S M, M M, et al. Cardiopulmonary bypass, steroid administration, and surgical injury synergistically impair memory T cell function and antigen presentation. *Interactive cardiovascular and thoracic surgery* 2003; 2(4): 598-602.

66 Sano T, Morita S, Masuda M, Yasui H. Minor Infection Encouraged by Steroid Administration during Cardiac Surgery. *Asian cardiovascular & thoracic annals* 2006; 14(6): 505-10.

67 Schurr UP, Zünd G, Hoerstrup SP, et al. Preoperative administration of steroids: influence on adhesion molecules and cytokines after cardiopulmonary bypass. *Annals of Thoracic Surgery* 2001; 72(4): 1316-20.

68 Sobieski MA, 2nd, Graham JD, Pappas PS, Tatooles AJ, Slaughter MS. Reducing the effects of the systemic inflammatory response to cardiopulmonary bypass: can single dose steroids blunt systemic inflammatory response syndrome? *ASAIO journal* (American Society for Artificial Internal Organs : 1992) 2008; 54(2): 203-6.

69 D S, MR K, M G, D S. Morbidity associated with systemic corticosteroid preparation for coronary artery bypass grafting in patients with chronic obstructive pulmonary disease: a case control study. *Journal of cardiothoracic surgery* 2007; 2: 25.

70 Suominen PK, Keski-Nisula J, Ojala T, et al. Stress-Dose Corticosteroid Versus Placebo in Neonatal Cardiac Operations: a Randomized Controlled Trial. *Annals of thoracic surgery* 2017; 104(4): 1378‐85.

71 Jahnukainen T, Keski-Nisula J, Tainio J, et al. Efficacy of corticosteroids in prevention of acute kidney injury in neonates undergoing cardiac surgery-A randomized controlled trial. *Acta anaesthesiologica Scandinavica* 2018; 00: 1-8.

72 Taleska Stupica G, Sostaric M, Bozhinovska M, et al. Extracorporeal Hemadsorption versus Glucocorticoids during Cardiopulmonary Bypass: A Prospective, Randomized, Controlled Trial. *Cardiovascular therapeutics* 2020; 2020: 7834173.

73 P T, JA R, A B, et al. Does high-dose methylprednisolone in aprotinin-treated patients attenuate the systemic inflammatory response during coronary artery bypass grafting procedures? *Journal of cardiothoracic and vascular anesthesia* 1999; 13(2): 165-72.

74 Teoh KHT, Bradley CA, Gauldie J, Burrows H. Steroid Inhibition of Cytokine-Mediated Vasodilation After Warm Heart Surgery. *Circulation* 1995; 92(9 Suppl): II347-53.

75 P T, K C, E T, CH N, S L. Effect of methylprednisolone on the oxidative burst activity, adhesion molecules and clinical outcome following open heart surgery. *Scandinavian cardiovascular journal* : SCJ 1997; 31(5): 283-8.

76 Toledo-Pereyra LH, Lin CY, Kundler H, Replogle RL. Steroids in heart surgery: A clinical double-blind and randomized study. *American Surgeon* 1980; 46(3): 155-60.

77 A T, A C, K B, et al. The effects of aprotinin and steroids on generation of cytokines during coronary artery surgery. *Journal of cardiothoracic and vascular anesthesia* 2001; 15(5): 603-10.

78 Vallejo JL, Giménez-Fernández R, Mainer JL, Rivera R. Clinical analysis of the protective effect of methylprednisolone on the heart in anoxic arrest (random study). *Revista Espanola De Cardiologia* 1977; 30(6 Pt 2): 705-9.

79 T V, M S, L E, et al. Influence of aminosteroid and glucocorticoid treatment on inflammation and immune function during cardiopulmonary bypass. *Critical care medicine* 2001; 29(11): 2137-42.

80 T V, M S, L E, et al. Effects of different steroid treatment on reperfusion-associated production of reactive oxygen species and arrhythmias during coronary surgery. *Acta anaesthesiologica Scandinavica* 2003; 47(6): 667-74.

81 Von Spiegel T, Giannaris S, Wrigge H, Schorn B, Hoeft A. Effects of dexamethasone on extravascular lung water and pulmonary haemodynamics in patients undergoing coronary artery bypass surgery. *Anasthesiologie Intensivmedizin Notfallmedizin Schmerztherapie* 2001; 36(9): 545-51.

82 T vS, S G, GJ W, et al. Effects of dexamethasone on intravascular and extravascular fluid balance in patients undergoing coronary bypass surgery with cardiopulmonary bypass. *Anesthesiology* 2002; 96(4): 827-34.

83 Vukovic PM, Maravic-Stojkovic VR, Peric MS, et al. Steroids and statins: an old and a new anti-inflammatory strategy compared. *Perfusion* 2011; 26(1): 31-7.

84 S W, JL L, CH H, et al. Does steroid pretreatment increase endotoxin release during clinical cardiopulmonary bypass? *The Journal of thoracic and cardiovascular surgery* 1999; 117(5): 1004-8.

85 F W, E K, B R, et al. Stress doses of hydrocortisone reduce chronic stress symptoms and improve health-related quality of life in high-risk patients after cardiac surgery: a randomized study. *The Journal of thoracic and cardiovascular surgery* 2006; 131(2): 277-82.

86 F W, A B-F, G S, et al. Stress doses of hydrocortisone in high-risk patients undergoing cardiac surgery: effects on interleukin-6 to interleukin-10 ratio and early outcome. *Critical care medicine* 2009; 37(5): 1685-90.

87 RP W, E Y, J N, F F, M B, KH T. Pulse low dose steroids attenuate post-cardiopulmonary bypass SIRS; SIRS I. *The Journal of surgical research* 2006; 132(2): 188-94.

88 Whitlock RP, Devereaux PJ, Teoh KH, et al. Methylprednisolone in patients undergoing cardiopulmonary bypass (SIRS): a randomised, double-blind, placebo-controlled trial. *Lancet (London, England)* 2015; 386(10000): 1243‐53.

89 JP Y, NJ S, L H-H, et al. Dexamethasone decreases the incidence of shivering after cardiac surgery: a randomized, double-blind, placebo-controlled study. *Anesthesia and analgesia* 1998; 87(4): 795-9.

90 JP Y, NJ S, FK T, et al. Effects of single dose, postinduction dexamethasone on recovery after cardiac surgery. *The Annals of thoracic surgery* 2000; 69(5): 1420-4.

91 JP Y, MH B, SC E, et al. Effect of dexamethasone on atrial fibrillation after cardiac surgery: prospective, randomized, double-blind, placebo-controlled trial. *Journal of cardiothoracic and vascular anesthesia* 2007; 21(1): 68-75.

92 Amr YM, E E, H E-S. Effects of dexamethasone on pulmonary and renal functions in patients undergoing CABG with cardiopulmonary bypass. *Seminars in cardiothoracic and vascular anesthesia* 2009; 13(4): 231-7.

93 M Y, S E, H A, K S, OA S, M C. Effect of low-dose methyl prednisolone on serum cytokine levels following extracorporeal circulation. *Perfusion* 1999; 14(3): 201-6.

94 JADAD A. Assessing the quality of reports of randomized clinical trials: is blinding necessary? *Controlled Clinical Trials* 1996; 17.

**eFigure 1. Impact of corticosteroids on hyperglycemia requiring insulin infusion (adult)
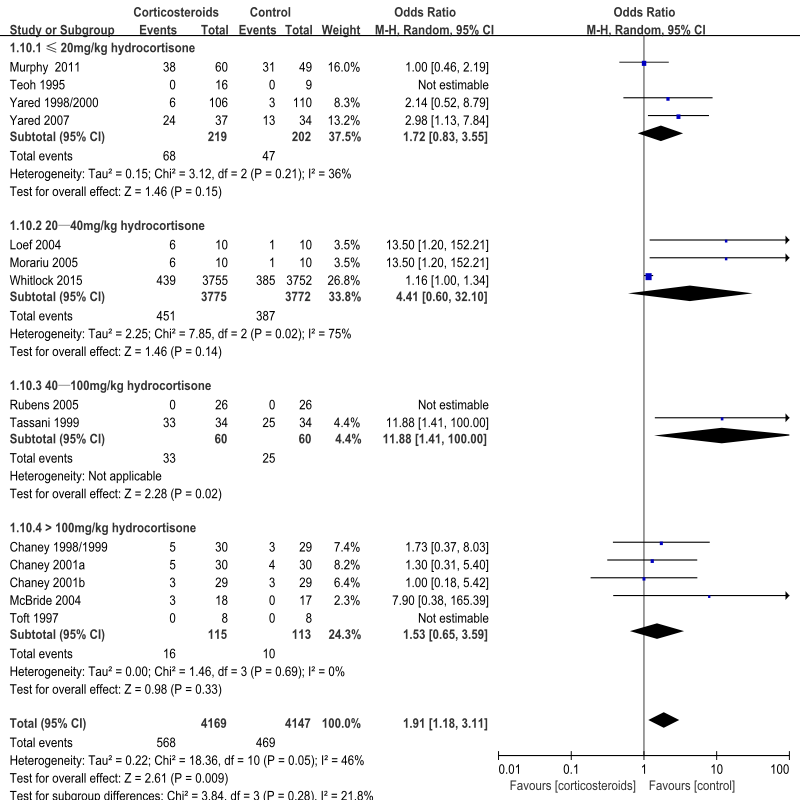
**

**eFigure 2. Impact of corticosteroids on LOS in ICU (days) (adult)
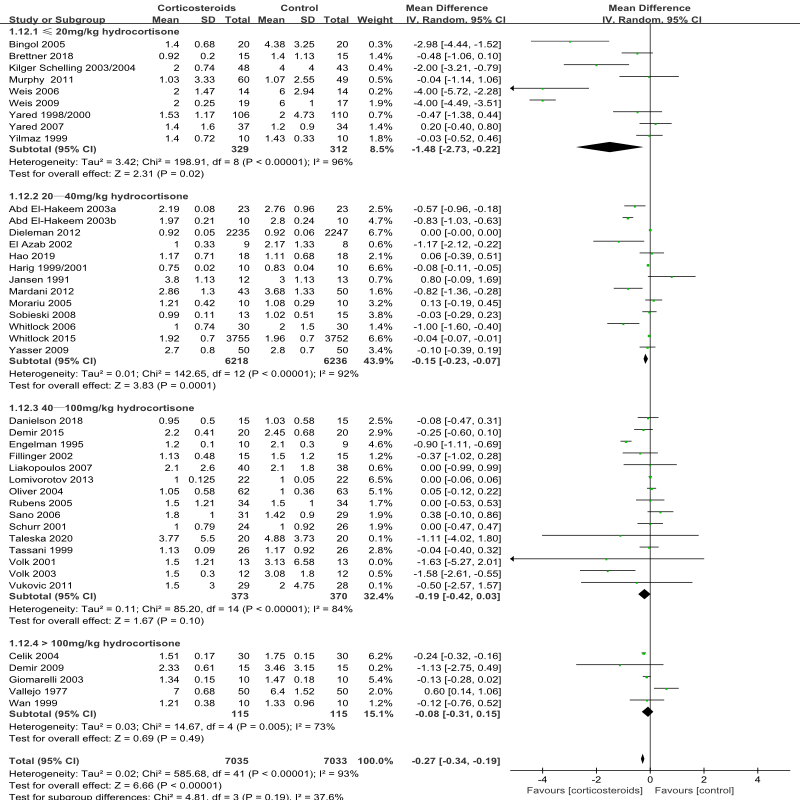
**

**eFigure 3. Impact of corticosteroids on LOS in hospital (days) (adult)
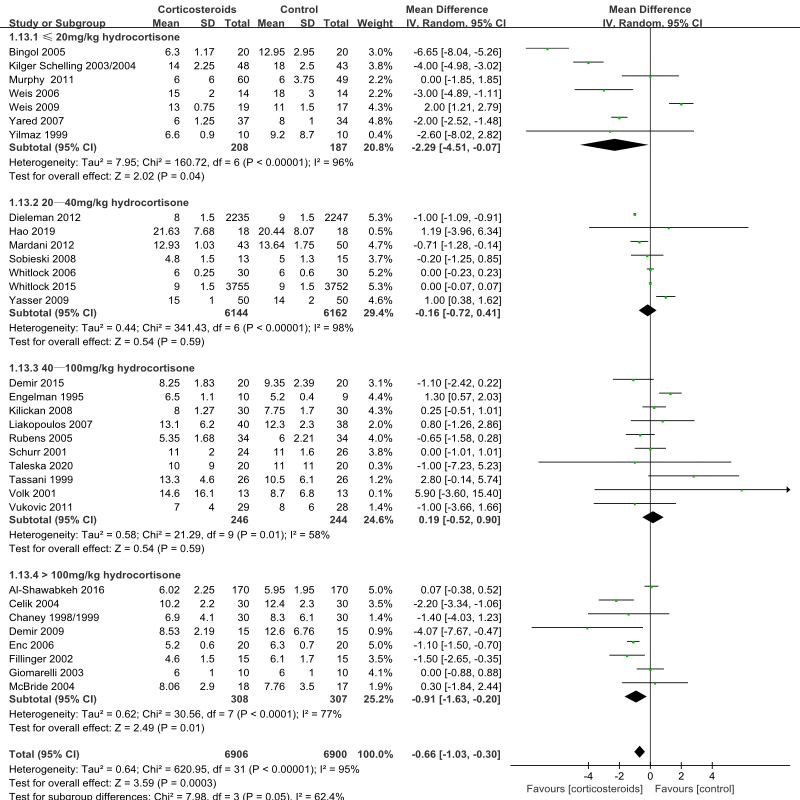
**

**eFigure 4. Impact of corticosteroids on postoperative bleeding (mL)(adult)
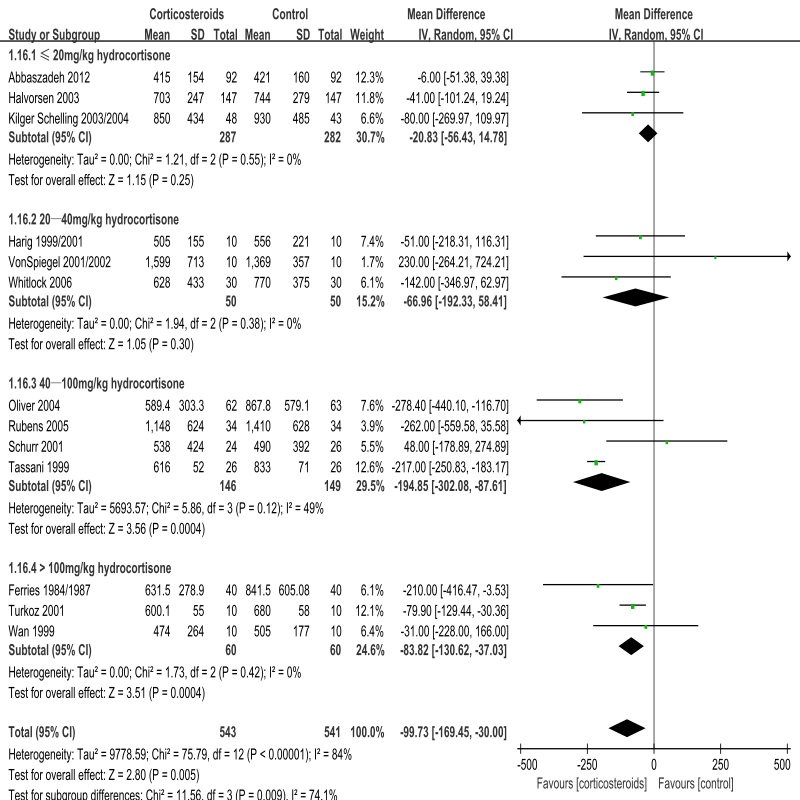
**

**eFigure 5. Impact of corticosteroids on re-intubation (adult)
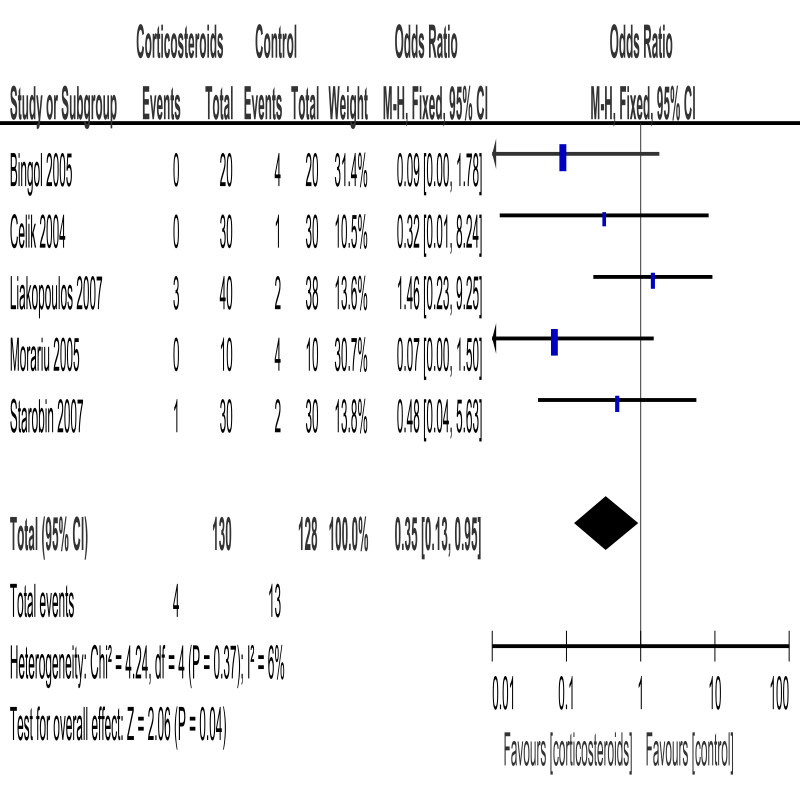
**

**eFigure 6. Impact of corticosteroids on IL-6 concentrations at 24 hours (pg/ml) (adult)
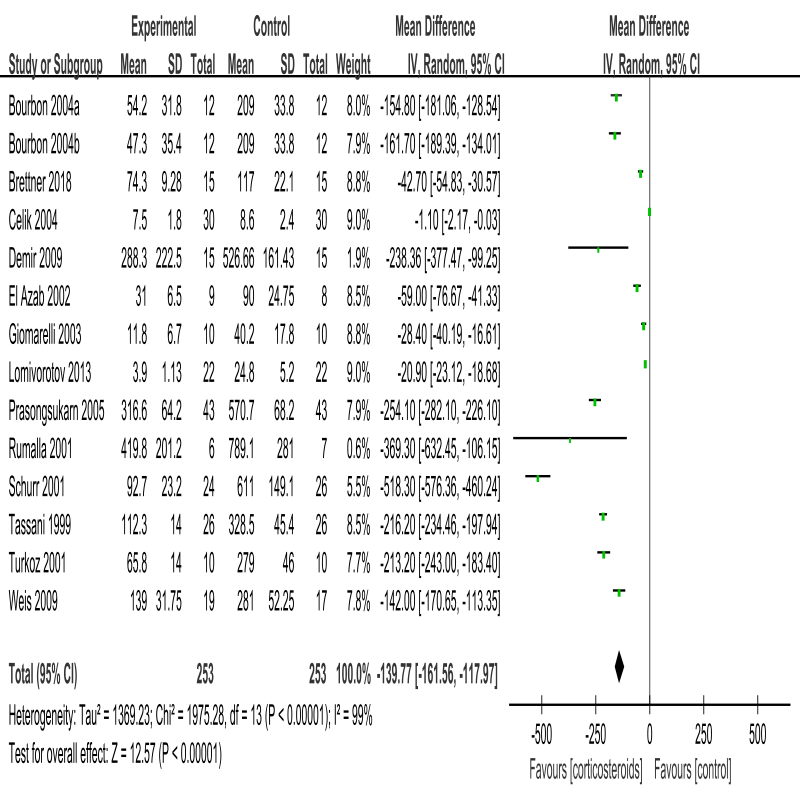
**

**eFigure 7. Impact of corticosteroids on TNF-****α concentrations at 24 hours (pg/ml) (adult)
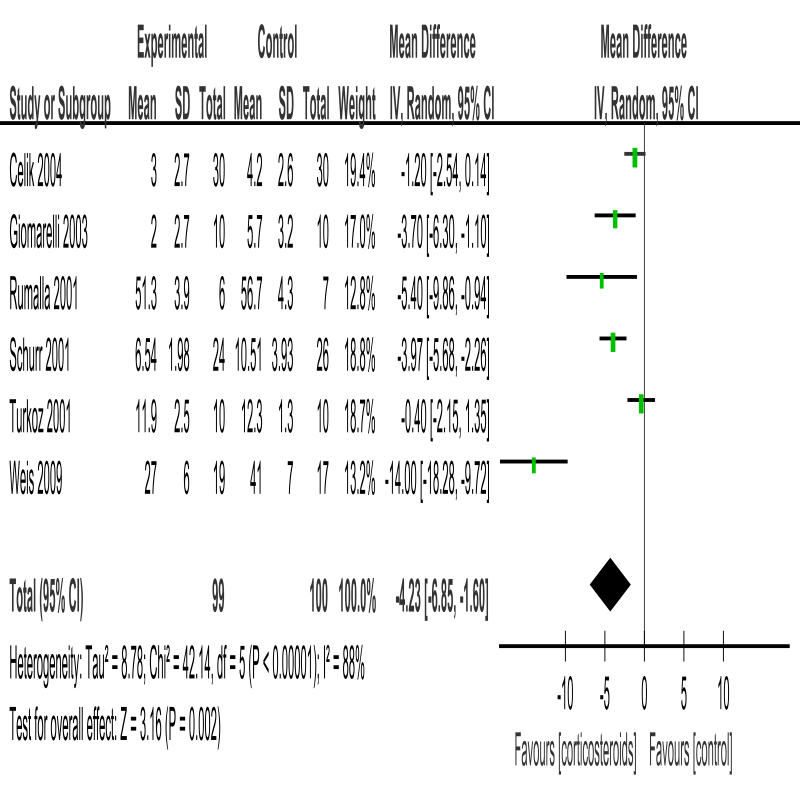
**

**eFigure 8. Impact of** **corticosteroids on IL-8 concentrations at 24 hours (pg/ml) (adult)
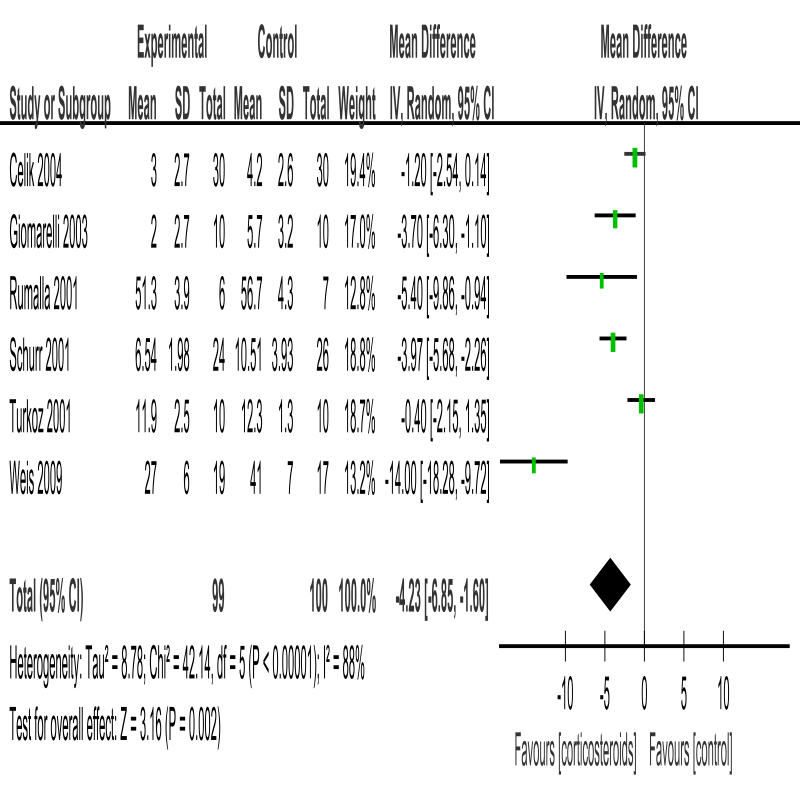
**

**eFigure 9. Impact of corticosteroids on highest/24h CRP concentrations (μg/ml)(pediatric)
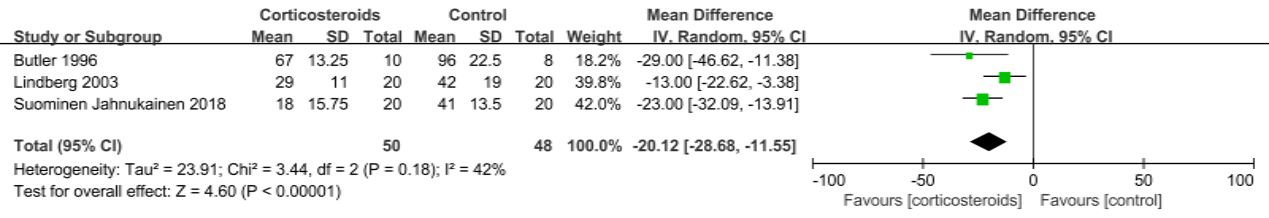
**

**eFigure 10. Impact of corticosteroids on highest/24h IL-6 concentrations (pg/ml) (pediatric)
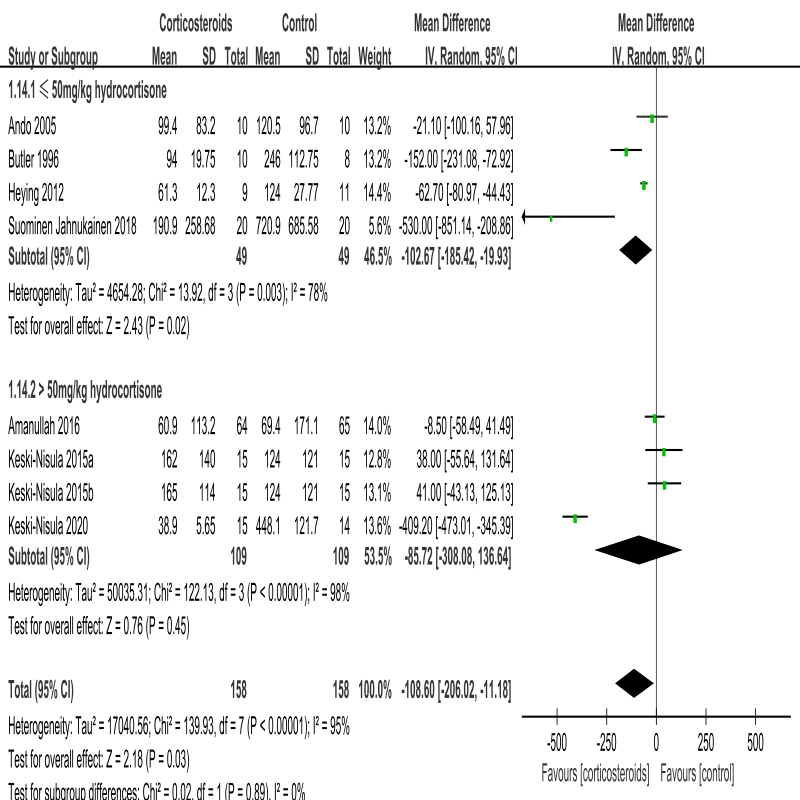
**

**eFigure 11. Impact of corticosteroids on highest/24h IL-10 concentrations (pg/ml) (pediatric)
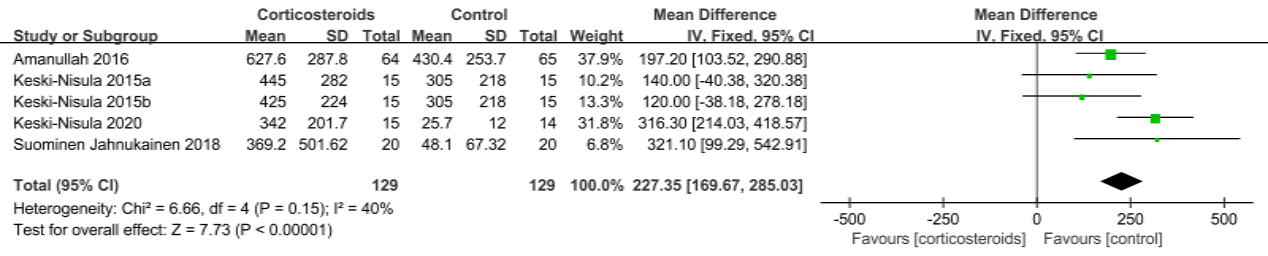
**

**eFigure 12. Impact of corticosteroids on kidney injury (adult)
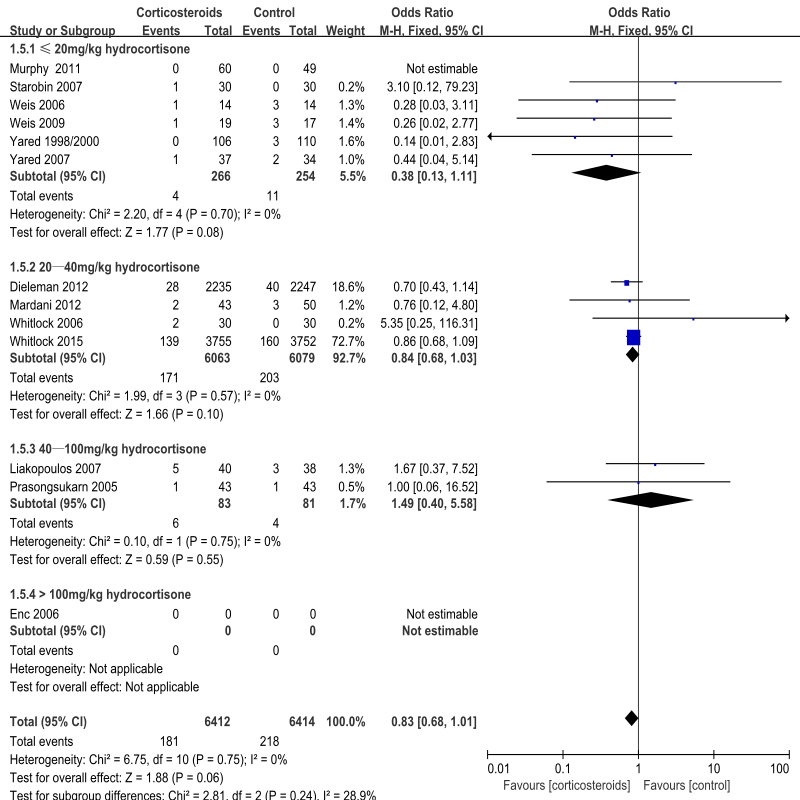
**

**eFigure 13. Impact of corticosteroids on pulmonary complications (adult)
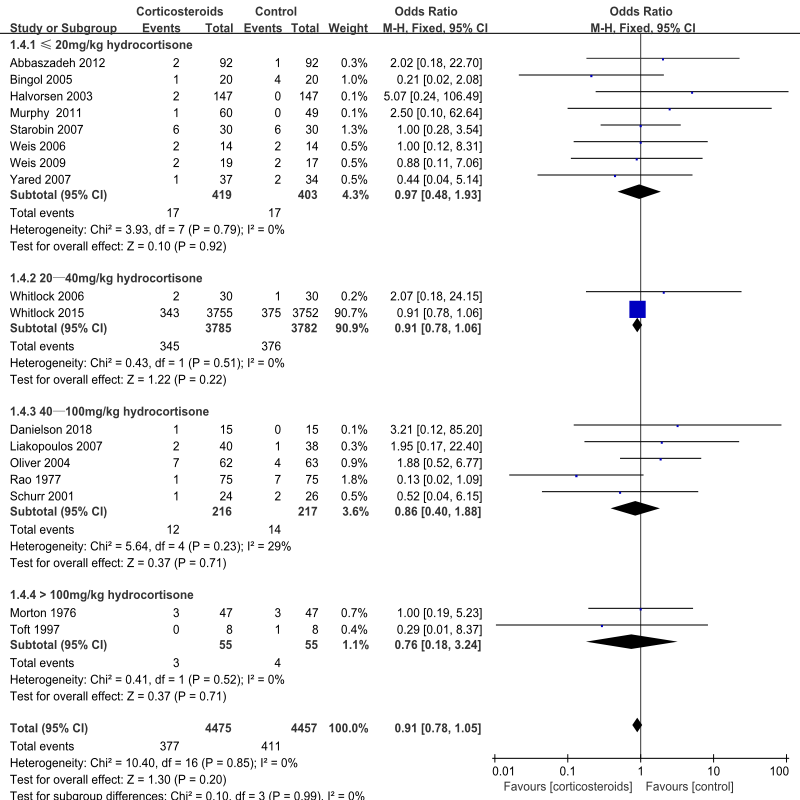
**

**eFigure 14. Impact of corticosteroids on neurological complications (strok)(adult)
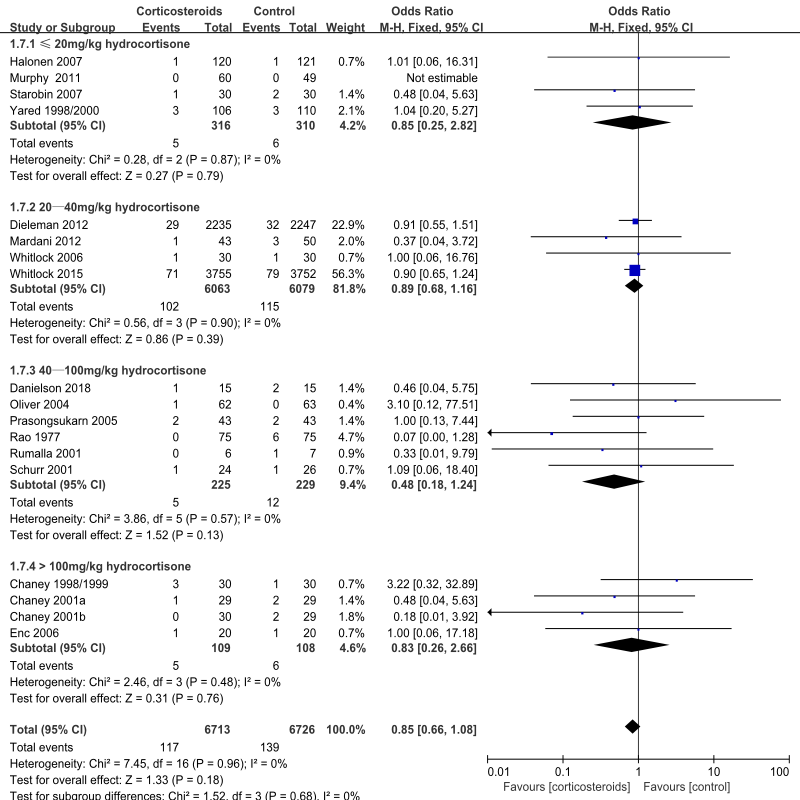
**

**eFigure 15. Impact of corticosteroids on gastro-intestinal bleeding (adult)
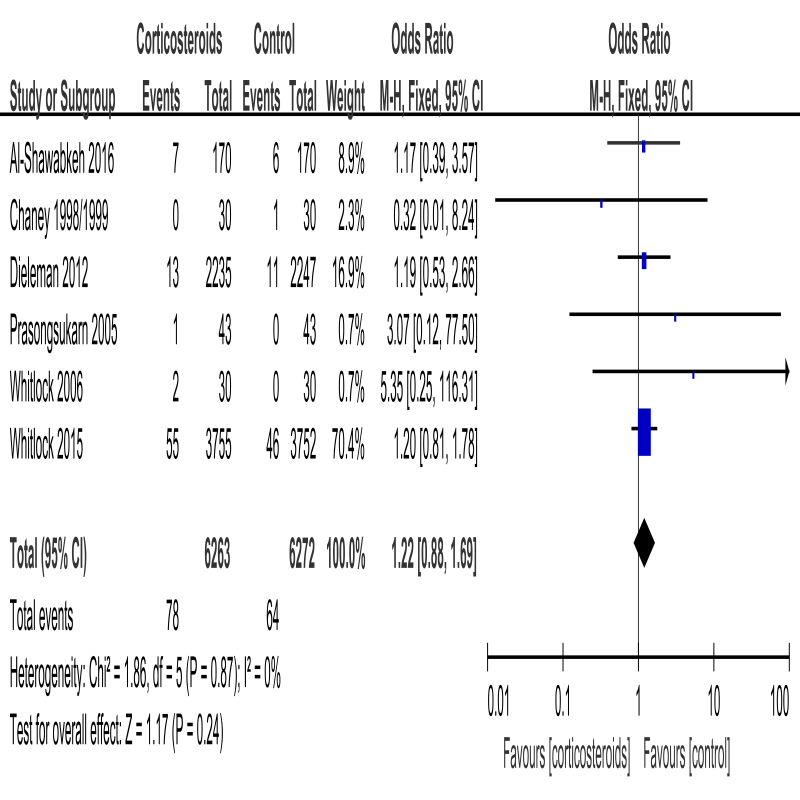
**

**eFigure 16. Impact of corticosteroids on postoperative infection (adult)
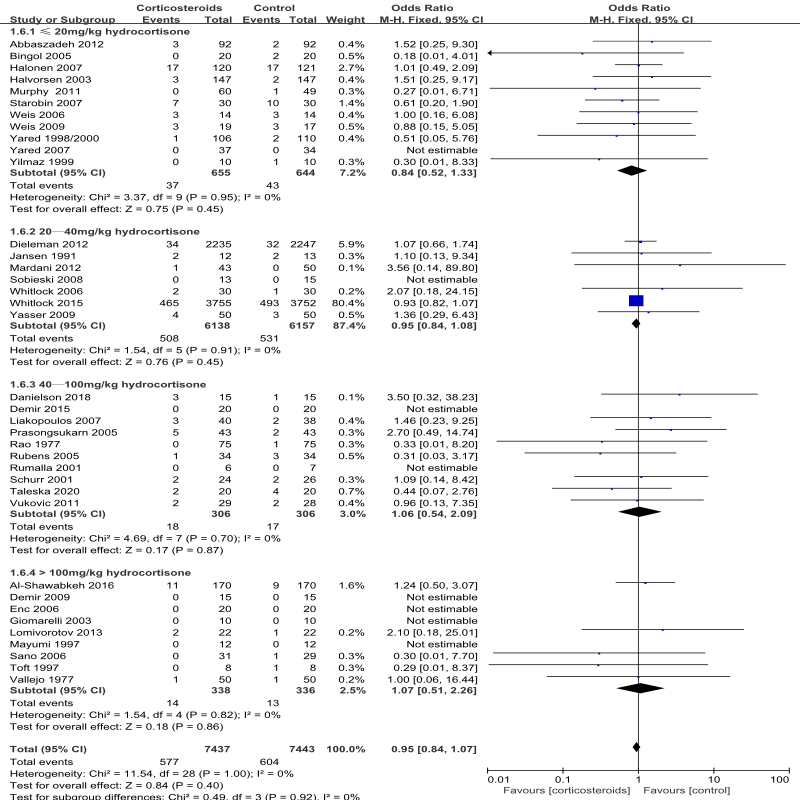
**

**eFigure 17. Impact of corticosteroids on delirium (adult)
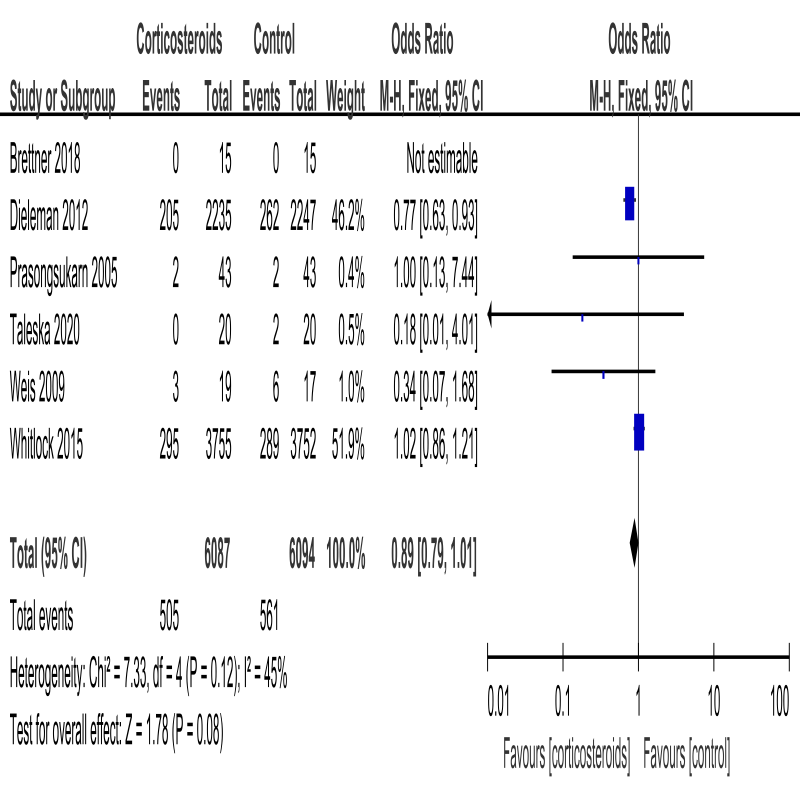
**

**eFigure 18. Meta-regression of doses of corticosteroids and reduction**

**in mortality risk for adult cardiac surgery with CPB**

**eFigure 19. Meta-regression of doses of corticosteroids and reduction**

**in new atrial fibrillation risk for adult cardiac surgery with CPB**

**eFigure 20. Meta-regression of doses of corticosteroids and reduction**

**in myocardial infarction risk for adult cardiac surgery with CPB**

**eFigure 21. Meta-regression of doses of corticosteroids and reduction**

**in pulmonary complications risk for adult cardiac surgery with CPB**

**eFigure 22. Meta-regression of doses of corticosteroids and reduction in kidney injury risk for adult cardiac surgery with CPB**

**eFigure 23. Meta-regression of doses of corticosteroids and reduction**

**in postoperative infection risk for adult cardiac surgery with CPB**

**eFigure 24. Meta-regression of doses of corticosteroids and reduction in neurological complications (strok) risk for adult cardiac surgery with CPB**

**eFigure 25. Meta-regression of doses of corticosteroids and reduction**

**in hyperglycemia requiring insulin infusion risk for adult cardiac surgery with CPB**

**eFigure 26. Meta-regression of doses of corticosteroids and reduction in LOS in ICU for adult cardiac surgery with CPB**

**eFigure 27. Funnel plot of mortality for adult cardiac surgery with CPB**

**eFigure 28. Funnel plot of myocardial infarction for adult cardiac surgery with CPB**

**eFigure 29. Funnel plot of pulmonary complications for adult cardiac surgery with CPB**

**eFigure 30. Funnel plot of kidney injury for adult cardiac surgery with CPB**

**eFigure 31. Funnel plot of postoperative infection for adult cardiac surgery with CPB**

**eFigure 32. Funnel plot of neurological complications (strok) for adult cardiac surgery with CPB**

**eFigure 33. Funnel plot of new atrial fibrillation for adult cardiac surgery with CPB**

**eFigure 34. Funnel plot of mechanical ventilation time for adult cardiac surgery with CPB**

**eFigure 35. Funnel plot of hyperglycemia requiring insulin infusion for adult cardiac surgery with CPB**

**eFigure 36. Impact of corticosteroids on duration of CPB (minutes) (pediatric)**


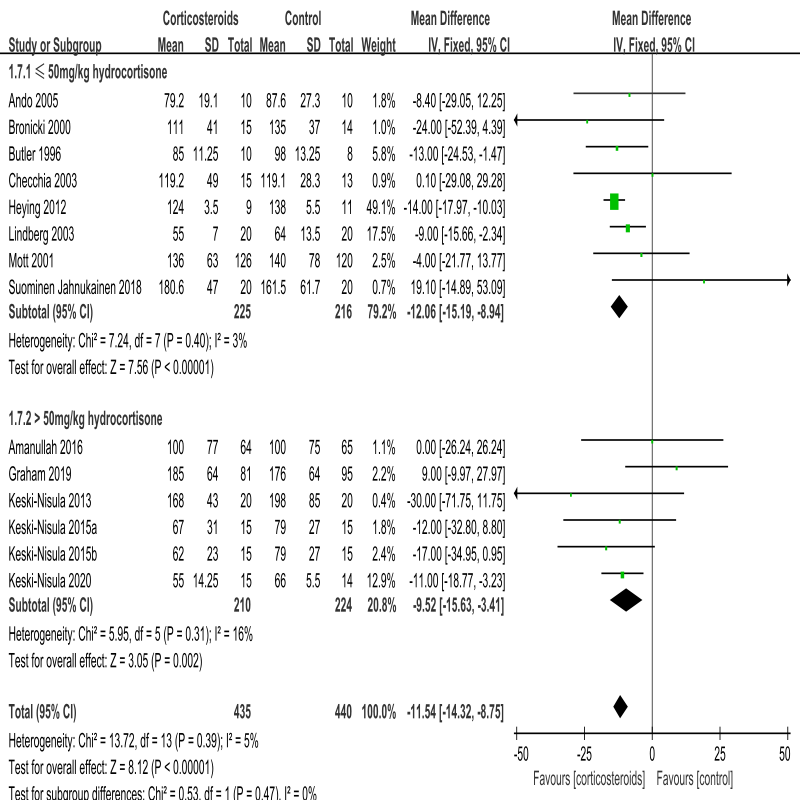


**eFigure 37. Impact of corticosteroids on hyperglycemia requiring insulin infusion (pediatric)
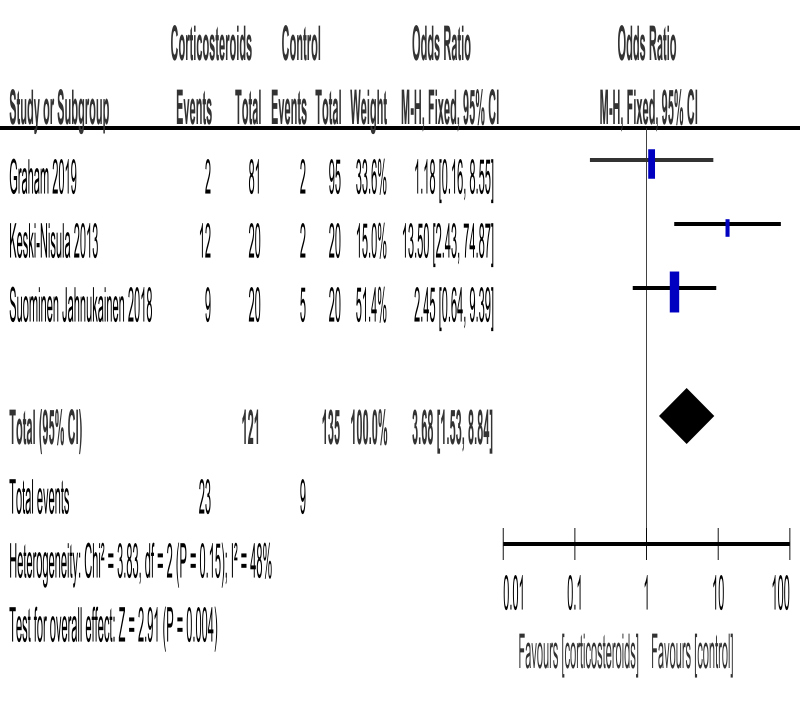
**

**eFigure 38. Impact of corticosteroids on mortality (pediatric)
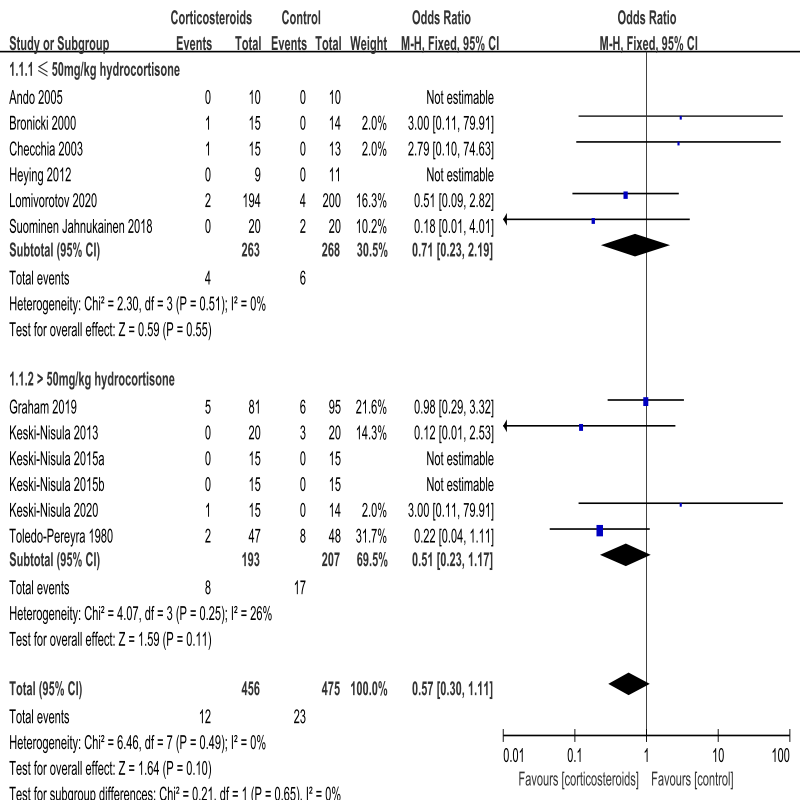
**

**eFigure 39. Impact of corticosteroids on kidney injury (pediatric)
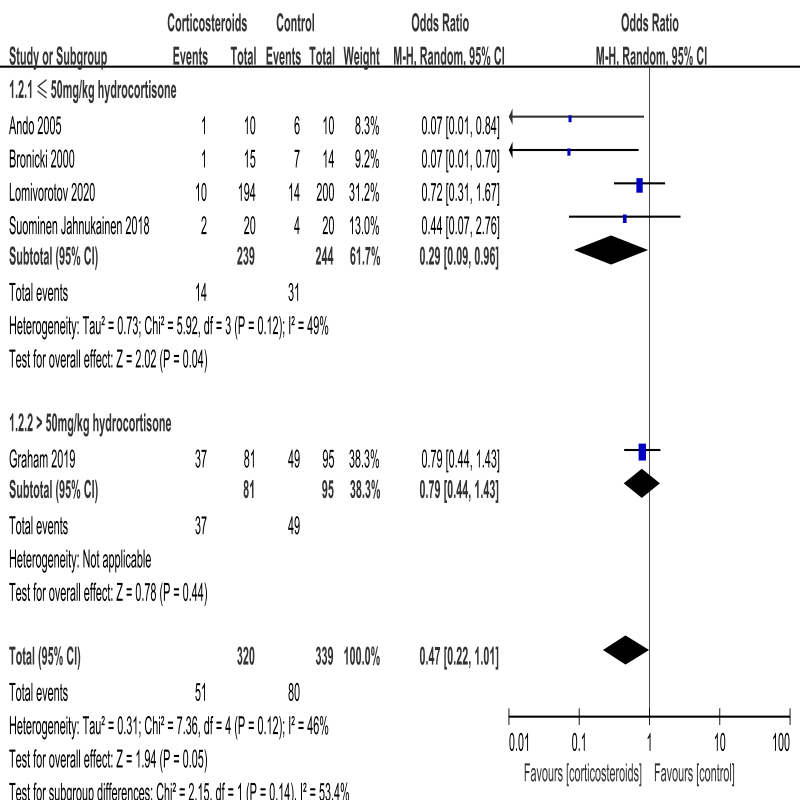
**

**eFigure 40. Impact of corticosteroids on postoperative ECMO use (pediatric)
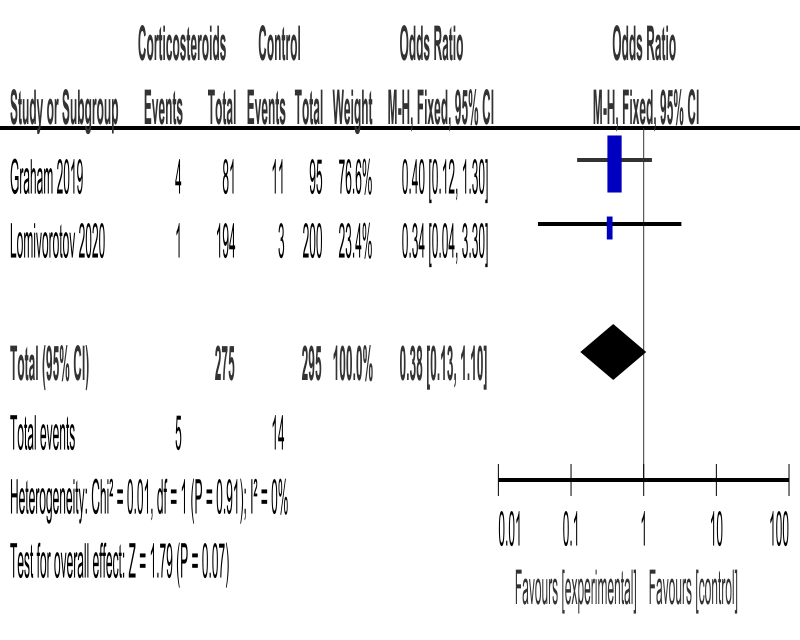
**

**eFigure 41. Impact of corticosteroids on postoperative infection (pediatric)
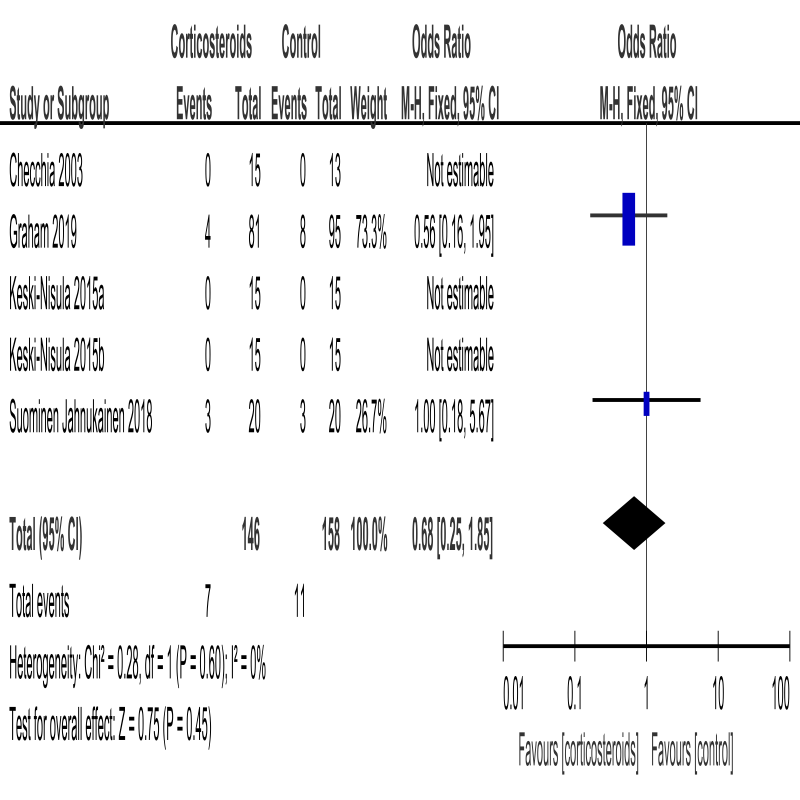
**

**eFigure 42. Impact of corticosteroids on mechanical ventilation time (pediatric)
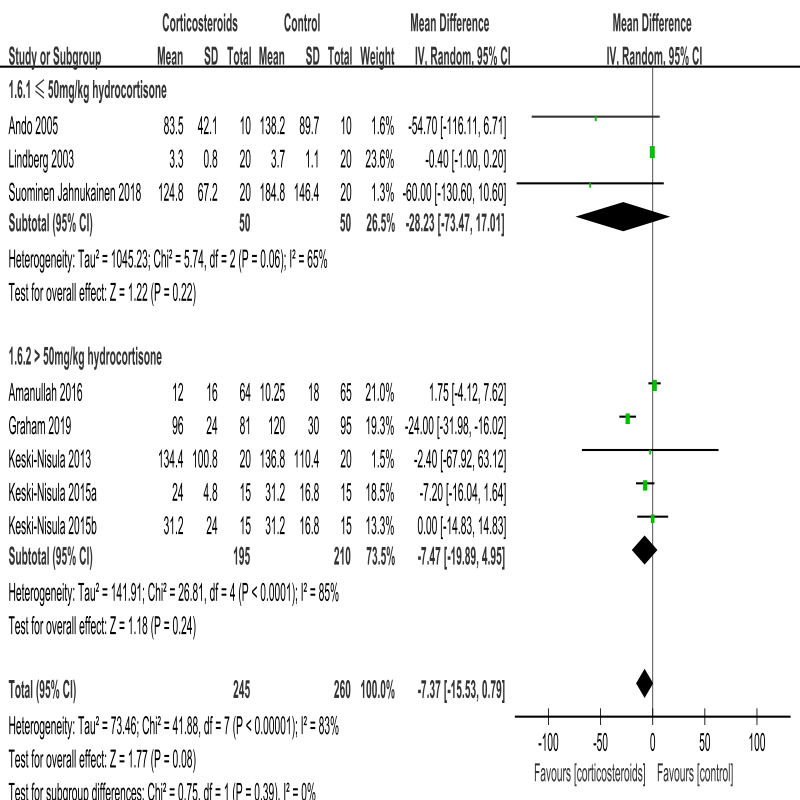
**

**eFigure 43. Impact of corticosteroids on LOS in ICU (pediatric)
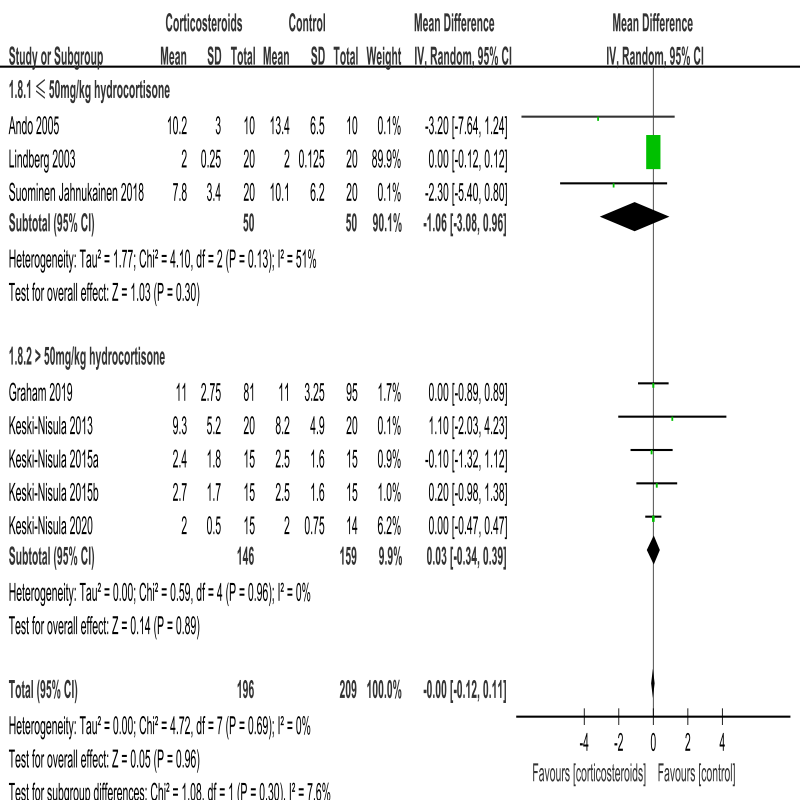
**

**eFigure 44. Meta-regression of doses of corticosteroids and reduction in mortality risk for pediatric cardiac surgery with CPB**

**eFigure 45. Meta-regression of doses of corticosteroids and reduction**

**in duration of CPB for pediatric cardiac surgery with CPB**

**eFigure 46. Funnel plot of mortality for pediatric cardiac surgery with CPB**

**eFigure 47. Funnel plot of kidney injury for pediatric cardiac surgery with CPB**

**eFigure 48. Funnel plot of postoperative infection for pediatric cardiac surgery with CPB**

**eFigure 49. Funnel plot of LOS in ICU for pediatric cardiac surgery with CPB**

**eFigure 50. Funnel plot of mechanical ventilation time for pediatric cardiac surgery with CPB**

**eFigure 51. Funnel plot of duration of CPB for pediatric cardiac surgery withCPB**
