# Supplementary material for: Meta-Analysis: Shouldn’t Prophylactic Corticosteroids be Administered During Cardiac Surgery with Cardiopulmonary Bypass?
Source: Front Surg. 2022 Jun 1;9:832205. doi: 10.3389/fsurg.2022.832205 (PMC9198450; doi:10.3389/fsurg.2022.832205)
Supplement: Supplementary file 2 [file Table_4_v1.doc]

| **Section/topic** | **#** | **Checklist item** | **Reported on page #** |
| --- | --- | --- | --- |
| **TITLE** | | |  |
| Title | 1 | Meta-analysis: shouldn't prophylactic corticosteroids be administered during cardiac surgery with cardiopulmonary bypass? | 1 |
| **ABSTRACT** | | |  |
| Structured summary | 2 | **Background:** Corticosteroid can effectively inhibit systemic inflammation induced by cardiopulmonary bypass. However, recently clinical trials and meta-analyses and guidelines do not support corticosteroid prophylaxis during cardiac surgery because of an increase in myocardial infarction and no benefit for patients. **Objectives:** We aimed to determine whether specific corticosteroid dose ranges might provide clinical benefits without increasing myocardial infarction. **Data sources:** The PubMed, Web of Science, Embase, ClinicalTrials, and Cochrane databases were searched for relevant randomized controlled trials (RCTs). **Study eligibility criteria:** RCTs that compared corticosteroids with a placebo or an equal volume of saline that was used before or at the beginning of CPB. **Participants:** Patients undergoing surgery with CPB for heart and/or valvular diseases were included. **Interventions:** Corticosteroid prophylaxis during cardiac surgery with cardiopulmonary bypass. **Study appraisal and synthesis methods:** This study was performed adhere to PRISMA guidelines. Random effect or fixed effect (I^2^<50%) models were used to evaluate the effects of corticosteroids prophylaxis. **Results:** 88 RCTs with 18,416 patients (17,067 adults and 1,349 children) were identified. Relative to placebo and high-dose corticosteroids, low-dose corticosteroids (≤20 mg/kg hydrocortisone) during adult cardiac surgery did not increase the risks of myocardial infarction or insulin infusion. However, low-dose corticosteroids were associated with lower risks of atrial fibrillation and kidney injury. Furthermore, low-dose corticosteroids significantly shortened the mechanical ventilation times, intensive care unit (ICU) stay, and hospital stay. During pediatric cardiac surgery, corticosteroids prophylaxis (≤50 mg/kg hydrocortisone) was associated with a reduced risk of kidney injury. **Limitations:** The low-dose subgroup for adult cardiac surgery (≤20 mg/kg hydrocortisone) only included 14 small RCTs, although 10 of these RCTs were considered high-quality based on the Jadad scores. **Conclusions and implications of key findings:** Low-dose corticosteroids prophylaxis during cardiac surgery provided significant benefits for adult patients, without increasing the risks of myocardial infarction and other complications. Corticosteroids prophylaxis (≤50 mg/kg hydrocortisone) reduced the risk of kidney injury in pediatric patients. **Systematic review registration number:** CRD42020193658 | 2,3,5,16 |
| **INTRODUCTION** | | |  |
| Rationale | 3 | CPB is used during most cardiac surgeries, although CPB often induces systemic inflammatory response syndrome (SIRS). The development of SIRS involves activation of complement, platelets, neutrophils, monocytes, macrophages, and cascade reactions, which leads to increased endothelial permeability, blood vessel damage, and parenchymal cell damage. These events are associated with single and multiple organ dysfunction, myocardial injury and infarction, respiratory failure, and ultimately death.  Corticosteroids are inexpensive drugs that can effectively inhibit inflammation, limit systemic capillary leak syndrome, and reduce organ damage, which provides a theoretical basis for their use during CPB. However, corticosteroids can cause side effects, including hyperglycemia, which is associated with immunosuppression and poor wound healing. In addition, high-dose corticosteroids are associated with an increased risk of gastrointestinal bleeding and myocardial infarction. Thus, the benefits of corticosteroids treatment are controversial for patients undergoing cardiac surgery with CPB.  Three meta-analyses of small RCTs revealed that prophylactic corticosteroids could reduce the risk of atrial fibrillation after adult cardiac surgery, also caused some side effects. Two large multi-center RCTs subsequently revealed that corticosteroids therapy provided no benefits and increased the risk of myocardial infarction in adult patients. Thus, the adult cardiac surgery guidelines do not recommend routine prophylactic use of corticosteroids during cardiac surgery, although there are no specific guidelines regarding corticosteroids use during pediatric cardiac surgery. We hypothesized that the specific corticosteroids dose range might influence the risks and benefits during cardiac surgery with CPB. | 3,4 |
| Objectives | 4 | This systematic review and meta-analysis aimed to evaluate the dose-dependent benefits and risks of prophylactic corticosteroids for adults and children undergoing cardiac surgery with CPB. | 4 |
| **METHODS** | | |  |
| Protocol and registration | 5 | The study protocol followed the PRISMA-P guidelines (<https://www.crd.york.ac.uk/prospero/display_record.php?ID=CRD42020193658>). PROSPERO registration number:: CRD42020193658 | 5 |
| Eligibility criteria | 6 | The meta-analysis only included RCTs that compared corticosteroids with a placebo used before or at the beginning of CPB. Studies were excluded if they used different concomitant medications or evaluated corticosteroids during off-pump heart surgery. Patients undergoing surgery with CPB for heart and/or valvular diseases were included. | 5 |
| Information sources | 7 | PubMed, Web of Science, Embase, ClinicalTrials, and Cochrane Central Register of Controlled Trials databases were searched for relevant RCTs that were published in any language before August 1, 2021. | 5 |
| Search | 8 | The search terms were: (“corticosteroids” OR “dexamethasone” OR “prednisolone” OR “prednisone” OR “methylprednisolone” OR “hydrocortisone”) AND (“cardiopulmonary bypass” OR “cardiac surgery”) AND (“randomized controlled trials”) | 5, eTable1 |
| Study selection | 9 | Two authors (TCC and XHZ) independently determined whether the identified articles fulfilled the inclusion criteria. There were no instances of disagreement regarding the extracted data. | 6 |
| Data collection process | 10 | The two authors also independently used pre-designed data extraction forms to record information regarding trial characteristics, clinical outcomes, randomization methods, application of blinding, allocation concealment, inclusion criteria, and exclusion criteria. There were no instances of disagreement regarding the extracted data. | 6 |
| Data items | 11 | Study characteristics included first author, publication date, country, study size, study design, randomization, blinding, follow-up duration, patient withdrawals, and study duration. Patient characteristics included age, sex, surgery type, blood pressure, history of diabetes, history of smoking, renal status, and fulfillment of the inclusion criteria. The interventions included the corticosteroids type, dose, timing, and route of administration during CPB. The outcomes included mortality, new atrial fibrillation, myocardial infarction, pulmonary complications (pulmonary edema), acute kidney injury, postoperative infection, neurological complications (stroke), delirium, gastrointestinal bleeding, extracorporeal membrane oxygenation (ECMO) use, mechanical ventilation time, insulin use, lengths of ICU and hospital stays, duration of CPB and procedure, postoperative bleeding, vasoactive medication use, re-thoracotomy, re-intubation, inotropic score, blood transfusion, and blood concentrations of glucose, lactate, C-reactive protein (CRP), tumor necrosis factor-α (TNF-α), interleukin (IL)-6, IL-8, and IL-10 at 24 h after CPB. | 6 |
| Risk of bias in individual studies | 12 | The Cochrane Handbook for Systematic Reviews of Interventions and Jadad score were used to assess the risk of bias for each trial. The two authors independently evaluated the risks of bias using the following domains: random sequence generation (selection bias), allocation concealment (selection bias), blinding of participants and personnel (performance bias), blinding of outcome assessment (detection bias), incomplete outcome data (attrition bias), selective outcome reporting (reporting bias), and other biases. The risk of bias in each domain was assessed as high, low, or uncertain. | 6 |
| Summary measures | 13 | The Mantel-Haenszel method was used to pool binary data and the results were reported as ORs with 95% CIs. An inverse variance analysis method was used to pool continuous data and the results were reported as MDs with 95% CIs. | 6 |
| Synthesis of results | 14 | The data were synthesized using Review Manager (version 5.3) and Stata software (version 16). Inter-study heterogeneity was assessed using the chi-squared test and I2 statistic, with the random effects model used for data with high heterogeneity (p<0.1 or I2>50%) and the fixed effects model used for data with less heterogeneity. | 6 |

Page 1 of 2

| **Section/topic** | **#** | **Checklist item** | **Reported on page #** |
| --- | --- | --- | --- |
| Risk of bias across studies | 15 | Risk of bias across studies were evaluated using the following domains: random sequence generation (selection bias), allocation concealment (selection bias), blinding of participants and personnel (performance bias), blinding of outcome assessment (detection bias), incomplete outcome data (attrition bias), selective outcome reporting (reporting bias), and other biases. The risk of bias in each domain was assessed as high, low, or uncertain. | 6 |
| Additional analyses | 16 | In this study, Sensitivity analysis was conducted to evaluate the robustness and the reliability of aggregation results by eliminating trials with high bias risk and the method of fill and trim was used to analyze publication bias. A meta-regression was performed to analyze the correlation between the dose of corticosteroids prophylaxis and clinical outcomes (pre-specified). A subgroup analysis was performed according to the dose of corticosteroids prophylaxis (not pre-specified). | 6 |
| **RESULTS** | | |  |
| Study selection | 17 | Records identified through database searching (n = 4915 ) and additional records identified through other sources (n = 63 ). Records after duplicates removed (n = 1137 ) and records screened (n = 1137 ). Records excluded (n = 1002 ) and full-text articles assessed for eligibility (n = 135 ). 41 articles excluded (11 no relevant outcomes, 8 non-RCTs, 9 no placebo, 7 no statement about treatment, 5 for animal study, 1 review). | Figure1 |
| Study characteristics | 18 | Study characteristics included first author, publication date, country, study size, study design, randomization, blinding, follow-up duration, patient withdrawals, and study duration. Patient characteristics included age, sex, surgery type, blood pressure, history of diabetes, history of smoking, renal status, and fulfillment of the inclusion criteria. The interventions included the corticosteroids type, dose, timing, and route of administration during CPB. | 7, Table1 |
| Risk of bias within studies | 19 | A total of 88 randomized controlled studies were included in this study, of which 26 jadad score scores were low quality and 62 were high quality. | Table2 |
| Results of individual studies | 20 | This meta-analysis revealed that corticosteroids prophylaxis during cardiac surgery with CPB was associated with significantly decreased blood inflammatory factor concentrations of CRP, TNF-α, IL-6, and IL-8. During adult cardiac surgery, corticosteroids prophylaxis reduced the risks of postoperative atrial fibrillation and re-intubation, shortened the ICU and hospital LOSs, and reduced postoperative bleeding, although it was associated with increased risks of myocardial infarction and hyperglycemia requiring insulin infusion. Interestingly, the benefits among adult patients were largely attributable to low-dose corticosteroids use (≤20 mg/kg hydrocortisone), as the benefits were not observed among patients who received higher corticosteroids doses. In addition, low-dose corticosteroids significantly reduced the mechanical ventilation time without increasing the risks of myocardial infarction and insulin infusion, while high-dose corticosteroids were associated with increased risks of myocardial infarction and prolonged mechanical ventilation. During pediatric cardiac surgery, corticosteroids prophylaxis was associated with a shortened CPB time, an increased risk of insulin infusion, and no substantial changes in terms of mortality, kidney injury, ECMO use, postoperative infection, mechanical ventilation time, and ICU LOS. Moreover, corticosteroids prophylaxis (≤50 mg/kg hydrocortisone) significantly reduced the risk of kidney injury in pediatric patients. | 7-10, Table2,  Table3, Figure2-5,  eFigure1-17 |
| Synthesis of results | 21 | Corticosteroids prophylaxis reduced the blood concentrations of some inflammatory markers in adult patients, which included IL-6 (MD: –139.77 pg/mL, 95% CI: –161.56, –117.97 pg/mL; p<0.001, I2=99%), TNF-α (MD: –4.23 pg/mL, 95% CI: –6.85, –1.60 pg/mL; p=0.002, I2=88%), and IL-8 (MD: –5.81 pg/mL, 95% CI: –10.96, –0.66 pg/mL; p=0.003, I2=97%). Among children, corticosteroids prophylaxis was associated with a significantly lower peak CRP concentration (MD: –20.12 µg/mL, 95% CI: –28.68, –11.55 µg/mL; p<0.001, I2=42%), a significantly lower IL-6 concentration (MD: –108.60 pg/mL, 95% CI: –206.02, –11.18 pg/mL; p=0.03, I2=95%), and a significantly higher IL-10 concentration (MD: 227.35 pg/mL, 95% CI :169.67–285.03 pg/mL; p<0.001, I2=40%) .  During adult cardiac surgery with CPB, corticosteroids prophylaxis was associated with increased risks of myocardial infarction (OR: 1.19, 95% CI: 1.05–1.35; p=0.008, I2=0%) and insulin infusion (OR: 1.91, 95% CI: 1.18–3.11; p=0.009, I2=46%), with no obvious improvement in mortality (OR: 0.86, 95% CI: 0.71–1.03; p=0.10, I2=0%). However, corticosteroids prophylaxis reduced the risk of postoperative atrial fibrillation (OR: 0.68, 95% CI: 0.57–0.82; p<0.0001, I2=48%), shortened the ICU stay (MD: –0.27 days, 95% CI: –0.34, –0.19 days; p<0.001, I2=93%), and shortened the hospital stay (MD: –0.66 days, 95% CI: –1.03, –0.30 days; p=0.0003, I2 = 95%). In addition, corticosteroids prophylaxis was associated with reduced postoperative bleeding (MD: –99.73 mL, 95% CI: –169.45, –30.00 mL; p=0.005, I2=84%) and a reduced risk of re-intubation (OR: 0.35, 95% CI: 0.13–0.95; p=0.04, I2=6%). Relative to the placebo group, corticosteroids prophylaxis was not associated with significant improvements in terms of kidney injury (OR: 0.83, 95% CI: 0.68–1.01; p=0.06, I2=0%), pulmonary complications (OR: 0.91, 95% CI: 0.78–1.05; p=0.20, I2=0%), stroke (OR: 0.85, 95% CI: 0.66–1.08; p=0.18, I2=0%), gastrointestinal bleeding (OR: 1.22, 95% CI: 0.88–1.69; p=0.24, I2=0%), postoperative infection (OR: 0.95, 95% CI: 0.84–1.07; p=0.40, I2=0%), delirium (OR: 0.89, 95% CI: 0.79–1.01; p=0.08, I2=45%), or mechanical ventilation time (MD: –0.48 h, 95% CI: –1.04, 0.09 h; p=0.1, I2=94%).  Subgroup analysis that the benefits were largely attributable to the prophylactic use of low-dose corticosteroids (≤20 mg/kg hydrocortisone), and these benefits were not observed at higher corticosteroids doses . Low-dose corticosteroids prophylaxis was associated with a significantly reduced mechanical ventilation time (MD: –2.74 h, 95% CI: –4.14, –1.33 h; p=0.0001, I2=92%), without increased risks of myocardial infarction (OR: 0.96, 95% CI: 0.43–2.17; p=0.93, I2=0%) or insulin infusion (OR: 1.72, 95% CI: 0.83–3.55; p=0.15, I2=36%).  During pediatric cardiac surgery with CPB, corticosteroids prophylaxis was associated with a decreased CPB time (MD: –11.54 min, 95% CI: –14.32, –8.75 min; p<0.001, I2=5%) and an increased insulin infusion (OR: 3.68, 95% CI: 1.53–8.84; p=0.004, I2=48%), but did not significantly influence mortality (OR: 0.57, 95% CI: 0.30–1.11; p=0.1, I2=0%), kidney injury (OR: 0.47, 95% CI: 0.22–1.01; p=0.05, I2=46%), ECMO use (OR: 0.38, 95% CI: 0.13–1.10; p=0.07, I2=0%), postoperative infection (OR: 0.68, 95% CI: 0.25–1.85; p=0.45, I2=0%), mechanical ventilation time (MD: –7.37 h, 95% CI: –15.53, 0.79 h; p=0.08, I2=83%), and ICU LOS (MD: –0.00 days, 95% CI: –0.12, 0.11 days; p=0.96, I2=0%). Relative to placebo and higher dose corticosteroids (>50 mg/kg hydrocortisone), corticosteroids prophylaxis (≤50 mg/kg hydrocortisone) significantly reduced the risk of kidney injury (OR: 0.29, 95% CI: 0.09–0.96; p=0.04, I2=49%). | 7-10, Table2,  Table3, Figure2-5,  eFigure1-17  eFigure36-43 |
| Risk of bias across studies | 22 | Funnel plots failed to reveal evidence of publication bias regarding mortality, myocardial infarction, pulmonary complications, kidney injury, postoperative infection, and neurological complications (stroke). However, the funnel plots suggested that there might be some publication bias regarding new atrial fibrillation, mechanical ventilation time, and hyperglycemia requiring insulin infusion. Thus, we used the trim and fill method to adjust the analysis, which did not significantly alter the findings.(adult cardiac surgery with CPB)  The funnel plots failed to reveal evidence of publication bias regarding mortality, kidney injury, postoperative infection, and ICU LOS. However, the funnel plots suggested that there might be some publication bias regarding mechanical ventilation time and CPB duration. Thus, we used the trim and fill method to adjust the analysis, which did not significantly alter the findings.(pediatric cardiac surgery with CPB) | 9, 10, eFigure27-35  eFigure46-51 |
| Additional analysis | 23 | During adult cardiac surgery with CPB, subgroup analysis that the benefits were largely attributable to the prophylactic use of low-dose corticosteroids (≤20 mg/kg hydrocortisone), and these benefits were not observed at higher corticosteroids doses. Low-dose corticosteroids prophylaxis was associated with a significantly reduced mechanical ventilation time (MD: –2.74 h, 95% CI: –4.14, –1.33 h; p=0.0001, I2=92%), without increased risks of myocardial infarction (OR: 0.96, 95% CI: 0.43–2.17; p=0.93, I2=0%) or insulin infusion (OR: 1.72, 95% CI: 0.83–3.55; p=0.15, I2=36%). Pooled analysis with meta-regression revealed that corticosteroids dose was significantly related to the variation in the mechanical ventilation time (exp: 1.004, 95% CI: 1.002–1.006; p<0.0001), but not the variation in the other clinical outcomes. The trim and fill method was used to adjust the analysis, which did not significantly alter the findings.  During pediatric cardiac surgery with CPB, corticosteroids prophylaxis (≤50 mg/kg hydrocortisone) significantly reduced the risk of kidney injury (OR: 0.29, 95% CI: 0.09–0.96; p=0.04, I2=49%). Meta-regression revealed that corticosteroids dose was not related to the variations in mortality (exp: 0.998, 95% CI: 0.981–1.015; p=0.734) or the duration of CPB (exp: 1.000, 95% CI: 0.993–1.008; p=0.89). The trim and fill method was used to adjust the analysis, which did not significantly alter the findings. | 9, 10, Table2,  Table3, Figure2-5,  eFigure1-26,  eFigure39,  eFigure44-45 |
| **DISCUSSION** | | |  |
| Summary of evidence | 24 | Our results suggest that low-dose corticosteroids (≤20 mg/kg hydrocortisone) were not associated with a significant reduction in mortality, but might substantially benefit adult patients by inhibiting SIRS and reducing complications. Therefore, we recommend prophylactic administration of low-dose corticosteroids (≤20 mg/kg hydrocortisone) during adult cardiac surgery. However, the optimal dose range for corticosteroids prophylaxis during pediatric cardiac surgery is unclear, as we only identified a small number of related RCTs. Nevertheless, our results indicate that high-dose glucocorticoids did not provide any benefits and significantly increased insulin use, which may increase the risk of hyperglycemia and related complications. | 11,12 |
| Limitations | 25 | The low-dose subgroup for adult cardiac surgery (≤20 mg/kg hydrocortisone) only included 14 small RCTs, although 10 of these RCTs were considered high-quality based on the Jadad scores. Thus, large multi-center RCTs are needed as an additional source of evidence to clarify efficacy and optimal dose range for low-dose prophylactic corticosteroids during adult and pediatric cardiac surgery with CPB. | 12,13 |
| Conclusions | 26 | Low-dose corticosteroids prophylaxis during cardiac surgery provided significant benefits for adult patients, without increasing the risks of myocardial infarction and other complications. Corticosteroids prophylaxis (≤50 mg/kg hydrocortisone) reduced the risk of kidney injury in pediatric patients. The large multi-center RCTs are needed as an additional source of evidence to clarify efficacy and optimal dose range for low-dose prophylactic corticosteroids during adult and pediatric cardiac surgery with CPB. | 14,15,16 |
| **FUNDING** | | |  |
| Funding | 27 | This work was supported by the National Natural Science Foundation of China [U2005202], the Fujian Province Major Science and Technology Program [2018YZ001-1], the Natural Science Foundation of Fujian Province [2020J01998, 2020J02056], and the Fujian provincial health technology project [2019-ZQN-50]. | 16 |

*From:*  Moher D, Liberati A, Tetzlaff J, Altman DG, The PRISMA Group (2009). Preferred Reporting Items for Systematic Reviews and Meta-Analyses: The PRISMA Statement. PLoS Med 6(6): e1000097. doi:10.1371/journal.pmed1000097

For more information, visit: **www.prisma-statement.org**.

Page 2 of 2
